# Chemical Diversity and Antitumor Metabolites from Soft Coral-Derived Fungus *Aspergillus sclerotiorum* SCSIO 41031 via OSMAC Strategy

**DOI:** 10.3390/md24040128

**Published:** 2026-03-31

**Authors:** Juan Gao, Jieyi Long, Xiaoyan Pang, Xuefeng Zhou, Yonghong Liu, Bin Yang

**Affiliations:** 1 Guangdong Key Laboratory of Marine Materia Medica/State Key Laboratory of Tropical Oceanography, South China Sea Institute of Oceanology, Chinese Academy of Sciences, Guangzhou 510301, China; gaojuan24@mails.ucas.ac.cn (J.G.); anna.long@infinitus-int.com (J.L.); xypang@scsio.ac.cn (X.P.); xfzhou@scsio.ac.cn (X.Z.); 2 University of Chinese Academy of Sciences, 19 Yuquan Road, Beijing 100049, China; 3 Infinitus (China) Company Ltd., Guangzhou 510623, China

**Keywords:** natural products, *Aspergillus sclerotiorum*, OSMAC, antitumor activity

## Abstract

Microorganisms provide critical lead compounds for drug development, yet most biosynthetic gene clusters remain silent under standard culture conditions. The OSMAC strategy activates these clusters by adjusting cultivation parameters, thereby enabling the discovery of novel compounds from a single strain. Here, we applied OSMAC to explore the metabolic potential of the soft coral-derived fungus *Aspergillus sclerotiorum* SCSIO 41031. Three different culture media were employed for the large-scale fermentation process. After isolation by chromatography, the compounds were structurally characterized using NMR, MS, and X-ray single-crystal diffraction, and their absolute configurations were determined by electronic circular dichroism (ECD) calculations. In total, three new compounds, named 6,6′-diacetyl-1,1′-dihydroxy-3,3′-dimethoxydibenzyl ether (**1**), esterwortmannolol (**17**) and pestalpolyol I (**20**), along with 19 known compounds (**2**–**16**, **18**–**19** and **21**–**22**) were obtained. This study validates the efficacy of the OSMAC strategy and underscores that *A. sclerotiorum* SCSIO 41031 serves as a valuable resource for producing structurally diverse natural products with potent biological activities.

## 1. Introduction

The ocean harbors a rich diversity of marine organisms, including cone snails, bacteria, cyanobacteria, fungi, and halophytic plants. These organisms not only contribute to more than 90% of the global biomass but also produce a wealth of structurally unique chemical compounds as a result of their evolutionary adaptation to extreme environments, offering valuable resources for drug discovery [1]. Marine-derived natural products have become increasingly important in drug discovery, showing remarkable promise especially in cancer treatment research. They represent an underexplored yet highly promising reservoir that warrants further investigation [2,3]. Unfortunately, the presence of microbial “silent gene clusters” often leads to either complete absence or only weak expression of secondary metabolites under standard laboratory cultivation conditions [4,5]. The one strain-many compounds (OSMAC) strategy has been demonstrated to be a simple yet powerful approach for activating silent gene clusters in microorganisms by modulating physicochemical culture parameters to regulate secondary metabolism and thereby promote the production of a broader spectrum of structurally diverse compounds [6]. This strategy has been successfully applied in numerous cases. For example, Yao et al. employed the OSMAC approach to further isolate three new cyclopentenone derivatives, aspergispones A-C, and five new cyclohexenone derivatives, aspergispones D-H, from the marine fungal strain *Aspergillus* sp. SCSIO 41501 [7]. Wang et al. subjected the marine-derived fungus *Ascotricha* sp. ZJ-M-5 to OSMAC analysis, and isolated three new caryophyllene derivatives from an oligotrophic medium [8]. Zhang et al. employed the OSMAC strategy to investigate the metabolome of the marine algae-derived strain *Streptomyces thermolineatus* NAK03196, guided by LC-MS profiling, and discovered a novel class of compounds featuring an unprecedented carbon skeleton, named chlorobactine A [9].

**Figure 1:**
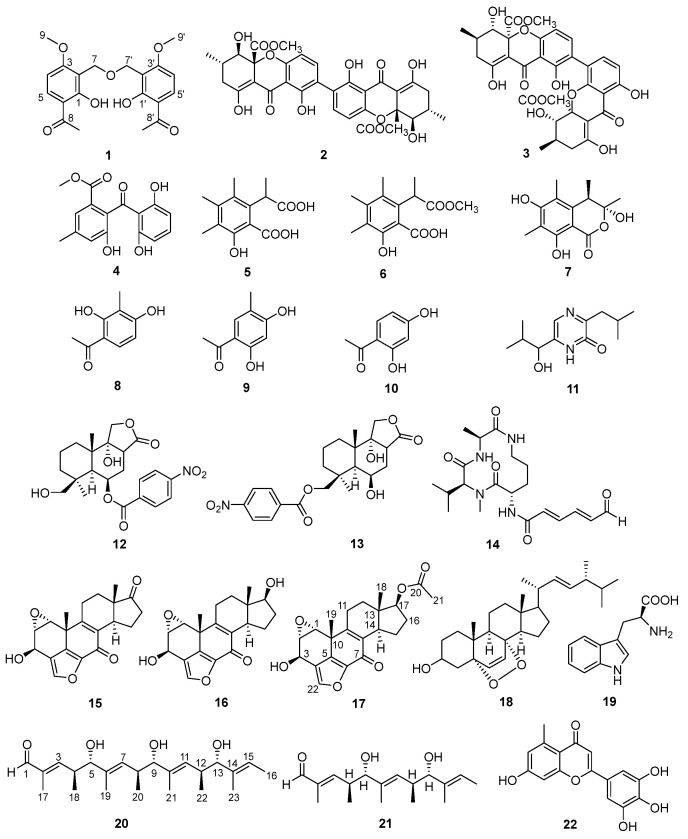
Structures of compounds isolated by the OSMAC approach.

In an ongoing study aimed at discovering structurally novel and biologically active natural products from marine fungi, we isolated three new cyclic peptide compounds from the strain *Aspergillus sclerotiorum* SCSIO 41031, namely three new cyclic hexapeptides, sclerotides C–E, and one new lipodepsipeptide, scopularide I, together with a known cyclic hexapeptide sclerotide A. Scopularide I exhibited cytotoxicity against the human nasopharyngeal carcinoma cell line HONE-EBV, with an IC_50_ value of 10.1 µM, and showed inhibitory activity toward acetylcholinesterase, with an IC_50_ value of 15.6 µM [10]. To explore additional bioactive compounds, a high-salt and oligotrophic OSMAC strategy was employed. This approach led to the isolation of two new secondary metabolites (**1** and **17**), one new natural product (**20**), along with 19 known compounds (**2**–**16**, **18**–**19**, and **21**–**22**) (Figure 1). Here, we report the isolation and structure elucidation of new metabolites discovered, together with the biological activities of these compounds.

## 2. Results

### 2.1. Isolation of Secondary Metabolites from Aspergillus Sclerotiorum SCSIO 41031 Through OSMAC

This study evaluated how cultivation conditions affect metabolite generation in *Aspergillus sclerotiorum* SCSIO 41031, using a systematic approach to isolate fermentation-derived compounds and elucidate their molecular structures. The data highlighted a pronounced sensitivity of metabolite diversity to environmental factors during cultivation. Firstly, fermentation was carried out in rice medium containing 3% (*w*/*v*) NaCl, from which 21 natural products, sclerotides A, C-E, scopularide I [10] and compounds (**1**–**16**), were isolated and structurally identified. Of these, compound **1** was characterized as a previously unreported structure, while the other 15 compounds were recognized as known substances through comparison with published data, supported by HRESIMS, 1D and 2D NMR, and optical rotation measurements. Known compounds **2**–**16** were identified as secalonic acid D (**2**) [11], penicillixanthone A (**3**) [12], 2,2′,6′-trihydroxy-4-methyl-6-methoxy-acyl-diphenylmethanone (**4**) [13], sclerin diacid (**5**) [14], sclerin diacid monoester (**6**) [14], (3R,4S)-6,8-dihydroxy-3,4,7-trimethylisocoumarin (**7**) [15], 2,4-dihydroxy-3-methylacetophenone (**8**) [16], 2,4-dihydroxy-5-methylacetophenone (**9**) [17], 4-acetyl-resorcinol (**10**) [18], 6-(1-hydroxy-2-methylpropyl)-3-(2-methylpropyl)-2(1H) pyrazinone (**11**) [19], insulicolide A (**12**) [20], 6β,9α-dihydroxy-14-p-nitrobenzoylcinnamolide (**13**) [20], pre-sclerotiotide F (**14**) [21], wortmannolone (**15**) [22], wortmannolol (**16**) [23].

Subsequently, the effect of rice medium containing 10% (*w*/*v*) NaCl was investigated. Under this condition, five natural products (**15**–**19**) were isolated, including one new compound (**17**), the remaining ones were identified by comparison with literature data. Compounds **18**–**19** were identified as 5α,8α-epidioxy-(22E,24R)-ergosta-6,22-dien-3β-ol (**18**) [24] and L-Tryptophan (**19**) [25].

Furthermore, to further investigate the effect of nutrient limitation, we carried out fermentation in an oligotrophic medium. Under these conditions, three natural products (**20**–**22**) were identified, including one new natural compound (**20**), the remaining compounds were identified as 5,9-dihydroxy-2,4,6,8,10-pentamethyldodeca-2,6,10-trienal (**21**) [26], penicillocitrin A (**22**) [27].

However, in our previous study, we identified novel bioactive peptide compounds from a rice-based culture medium. The selection of two additional fermentation conditions was intended to thoroughly investigate the strain’s capacity to produce peptide-like metabolites, particularly in the hope of discovering more cyclic peptide structures analogous to sclerotide. Unfortunately, no such compounds were isolated under either of the alternative conditions, suggesting that both salinity and fermentation duration may play a critical role in the biosynthesis of this class of molecules.

### 2.2. Structure Elucidation of Novel Compounds

**Figure 2:**
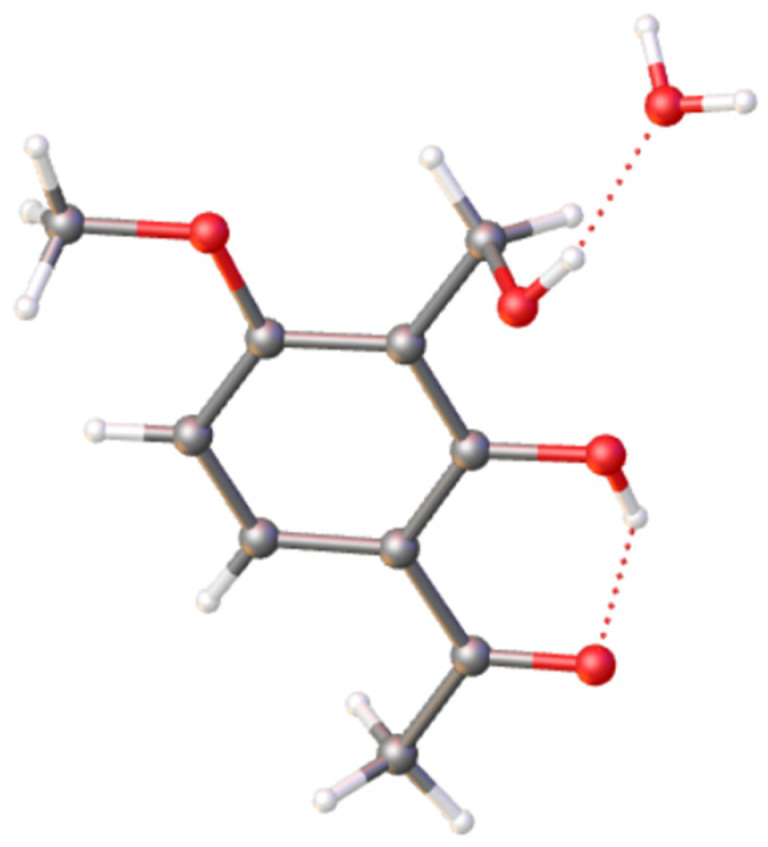
ORTEP drawing of the monomer structure of compound **1**.

**Figure 3:**
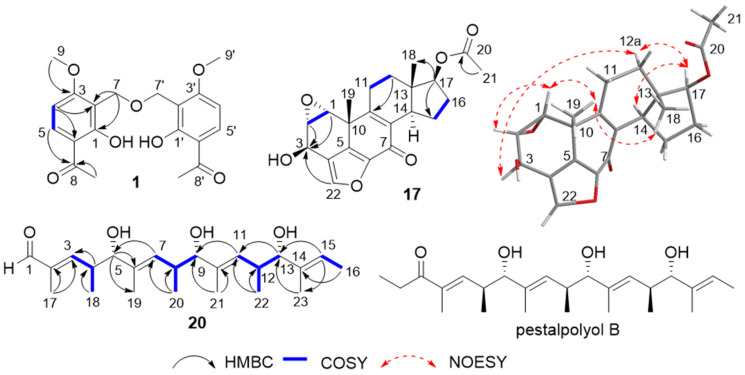
Key ^1^H−^1^H COSY and HMBC correlations of compounds **1**, **17**, **20** and key NOESY correlations of compound **17**.

Compound **1** was obtained as a pink powder. The ^13^C NMR data (Table 1) exhibited ten carbon signals, including two methyl groups, one sp^3^-hybridized methylene group, two sp^2^-hybridized methine groups, four sp^2^-hybridized quaternary carbons, and one carbonyl carbon. The ^1^H NMR spectrum showed two methyl groups [*δ*_H_ 3.94 (3H, s, 9-OCH_3_), *δ*_H_ 2.58 (3H, s, 10-CH_3_)], one oxygen-linked methylene group [*δ*_H_ 4.70 (2H, s, 7-CH_2_)], and two olefinic protons [*δ*_H_ 7.90 (1H, m, 5-CH), *δ*_H_ 6.64 (1H, m, 4-CH)]. Additionally, an exchangeable proton signal appeared at *δ*_H_ 12.89 (1H, s, 1-OH), indicative of hydrogen bonding and consistent with the presence of a CH_2_–O–CH_2_ linkage in a dibenzyl ether framework (Figures S1–S3). These data suggested a tetrasubstituted benzene ring bearing a carbonyl group. However, the HRESIMS data *m*/*z* 375.1433 [M + H]^+^ showed that the molecular formula is C_20_H_23_O_7_ with a degree of unsaturation of 10 (Figure S7). Therefore, it was speculated that the compound has a highly symmetric structure. Its monomeric structure was confirmed by single-crystal X-ray diffraction (Figure 2). In the HMBC (Figure 3 and Figure S6) of this compound, H-4 correlated with C-2 and C-6, H-5 correlated with C-8, H-9 correlated with C-3, and H-7 correlated with C-1 and C-2. In the ^1^H-^1^H COSY (Figure 3 and Figure S5), H-4 correlated with H-5, confirming the above speculations. These correlations established the planar structure shown in Figure 1. On the basis of its structural features, compound **1** was tentatively named 6,6′-diacetyl-1,1′-dihydroxy-3,3′-dimethoxydibenzyl ether.

**Figure 4:**
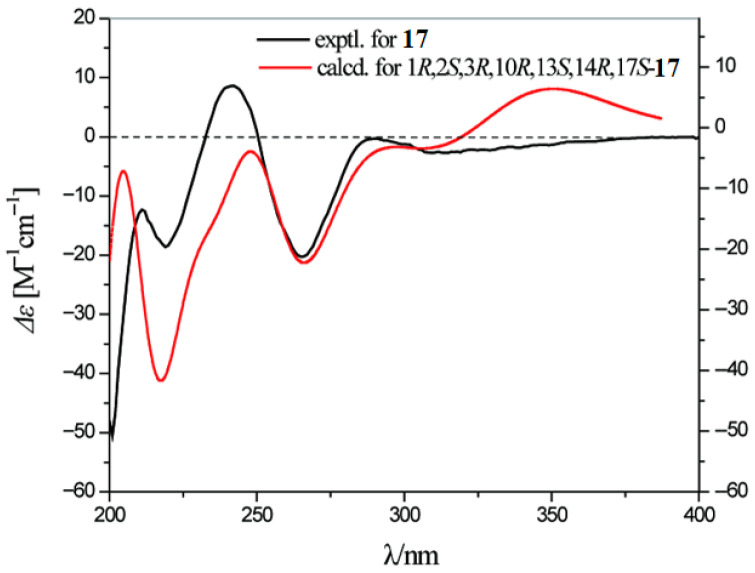
Experimental and calculated ECD spectra of compound **17**.

Compound **17** (esterwortmannolol) was isolated as a colorless powder. Combined HRESIMS (Figure S14) and ^13^C NMR (DEPT) data analysis (Table 2) determined the molecular formula as C_22_H_24_O_6_. The NMR data of compound **17** closely resembled those of wortmannolol (**15**) [23], the main differences being the presence of a carbonyl carbon signal at *δ*_C_ 176.2 (C-20) in the downfield region and a methyl carbon signal at *δ*_C_ 21.0 (C-21) in the upfield region (Figures S8 and S9). Furthermore, HMBC correlations (Figure 3 and Figure S12) showed a clear cross-peak between H-21 and C-20, which led to the conclusion that compound **17** is an acetyl derivative resulting from esterification at C-17. NOESY experiments (Figure 3 and Figure S13) revealed through-space correlations among H-1/H-19/H-18, H-3/H-12a/H-17/H-14, and H-1/H-2, supporting the relative configuration. Comparison of the calculated electronic circular dichroism (ECD) spectrum with the experimentally measured CD curve (Figure 4) enabled assignment of the absolute configuration as 1*R*, 2*S*, 3*R*, 10*R*, 13*S*, 14*R*, 17*S*.

Compound **20** (pestalpolyol I) was obtained as a white powder. HRESIMS (Figure S21) and ^13^C NMR (DEPT) analyses revealed a pseudo-molecular ion at *m*/*z* 401.2662 [M + Na]^+^, corresponding to the molecular formula C_23_H_38_O_4_. Based on 2D NMR data, including HSQC, HMBC and COSY (Figures S15–S19), the structure of compound **20** was established as a linear polyketide featuring multiple double bonds, hydroxyl groups, and polymethyl substituents [28]. The ^1^H NMR, COSY, and HMBC spectra (Figure 3) were compared with literature data and found to closely resemble those of pestalpolyol B [29], except that compound **20** lacks an ethyl group and contains an aldehyde functionality instead of a ketone (Table 3). The coupling constant of 7.9 Hz between the allylic protons H-4/H-5, H-8/H-9, and H-12/H-13 suggests a gauche-dominant conformation of the adjacent methylene groups. Large coupling constants (9.7 Hz, 9.2 Hz and 9.1 Hz) for H-3/H-4, H-7/H-8, and H-11/H-12 indicated trans (E) geometry of these double bonds. NOESY experiments revealed through-space correlations between H-3/H-18, H-7/H-20, and H-11/H-22 with H-5, H-9, and H-13, respectively, consistent with these protons residing on the same face of the molecule (Figure S20). The specific rotation [α${]}_{D}^{25}$ = +14.56 (*c* = 0.13, MeOH) paralleled the trend observed for pestalpolyols A–D. Based on the high similarity in NMR data (particularly the ^1^H and ^13^C chemical shifts) with the known compound pestalpolyol B, along with their extremely close specific optical rotation values, we propose that compound **20** possesses the same absolute configuration as pestalpolyol B at these chiral centers. On this basis, the double bond geometries were assigned as 2*E*, 6*E*, 10*E*, and 14*E*, and the absolute configurations at the stereogenic centers were determined as 4*S*, 5*S*, 8*S*, 9*S*, 12*S*, and 13*S*.

### 2.3. Bioassays

In vitro antitumor activity assays were performed for compounds **1**–**22** against a panel of cancer cell lines, including Hela, HL-60, K562, Molt-4, ACHN, 786-O, and OS-RC-2. Compound **22** displayed cytotoxicity against the Hela cell line, with an IC_50_ value of 50.02 μM. Compounds **12** and **13** have also shown significant activity against four of the cell lines (K562, Molt-4, Hela, and HL60) (Table 4, Figure S22), and the relevant data were reported in our earlier research paper [20]. At a concentration of 10 μM, several compounds displayed distinct biological activities. Compounds **1**, **5**, **6**, **8**, **15**, and **16** weakly protected primary cortical neurons from Aβ_25–35_-induced apoptosis. Meanwhile, in LPS-stimulated RAW264.7 macrophages, compounds **5**, **6**, and **13** demonstrated weak anti-inflammatory activity, as indicated by reduced nitric oxide (NO) production, while compounds **7**, **9**, and **15** exhibited weak pro-inflammatory effects.

## 3. Materials and Methods

### 3.1. General Experimental Procedures

Optical rotation values were determined using a MCP 500 automated polarimeter (Anton Paar, Graz, Austria), with methanol as the solvent. Ultraviolet-electronic circular dichroism (UV-ECD) spectra were acquired on a Chirascan CD spectrometer (Applied Photophysics Ltd., Surrey, UK). ^1^H and ^13^C NMR, along with DEPT and 2D NMR spectra, were recorded on a Bruker Avance 700 spectrometer (Billerica, MA, USA). HRESIMS measurements were performed using a Bruker maXis QTOF instrument; conventional ESIMS data were collected with a Bruker amaZon SL ion-trap mass spectrometer. Single-crystal X-ray diffraction intensity data were obtained on a CrysAlis PRO CCD area-detector diffractometer (Agilent, Santa Clara, CA, USA) employing graphite-monochromated Cu Kα radiation (λ = 1.54178 Å). For purification steps, TLC was carried out on pre-coated silica gel GF_254_ plates (particle size 10–40 µm). Column chromatography (CC) was conducted using silica gel (200–300 mesh, Qingdao Marine Chemical Factory, Qingdao, China). All solvents employed were of analytical grade and sourced from Qingdao Marine Chemical Factory (Qingdao, China). HPLC analyses were implemented on either a Hitachi Primaide system equipped with a YMC ODS column (YMC-Pack ODS-A, YMC Co. Ltd., 250 × 10 mm i.d., S-5 µm, 12.0 nm, 2.0 mL/min, Kyoto, Japan) or an Agilent 1260 S3 HPLC system equipped with an ODS column (YMC-Pack ODS-A, 250 × 4.6 mm i.d., S-5 µm, 12 nm, 1.0 mL/min).

### 3.2. Fungal Material

Strain SCSIO 41031 was isolated from a soft coral collected in Beihai, Guangxi Province, China. The isolate was maintained on a slanted agar plate of Müller-Hinton broth (MB) containing malt extract 15.0 g, artificial seawater salt 10.0 g, and agar 15.0 g, and stored at 4.0 °C. A voucher specimen has been placed in the CAS Key Laboratory of Tropical Marine Bio-Resources and Ecology, South China Sea Institute of Oceanology, Chinese Academy of Sciences, Guangzhou, China. The ITS1-5.8S-ITS4 sequence of the strain SCSIO 41031 was amplified by polymerase chain reaction (PCR) and sequenced. A BLAST 2.12.0 comparison with sequences deposited in GenBank revealed that 1070 base pairs shared 99% identity with *Aspergillus sclerotiorum* RSPG_179 (GenBank No. KC478520.1). Based on these results, strain SCSIO 41031 was identified as *Aspergillus sclerotiorum.*

### 3.3. Cultivation and Extraction

*Aspergillus sclerotiorum* strain SCSIO 41031 was initially cultured on MB agar plates at 25.0 °C for 7 days. A seed culture was prepared by inoculating the strain into seed medium (per liter of tap water: malt extract 15.0 g, artificial seawater salt 10.0 g; pH 7.4–7.8) and incubating at 25.0 °C on a rotary shaker (180 rpm) for 48 h. Large-scale fermentation in solid rice medium of normal salinity was performed as follows: at room temperature, strain SCSIO 41031 was inoculated into thirty 1-L conical flasks, each containing 200.0 g rice, 8.0 g artificial seawater salt, and 250 mL water. Fermentation was carried out for 30 days. The whole fermentation material was extracted three times with ethyl acetate to afford a brown extract (102 g). Large-scale fermentation in solid rice medium of high salinity: at room temperature, strain SCSIO 41031 was inoculated into forty-five 1-L conical flasks, each containing 200.0 g rice, 25.0 g artificial seawater salt, and 250 mL water. Fermentation was performed for 30 days. The fermentation material was extracted three times with ethyl acetate to afford a brown extract (16.5 g). Large-scale fermentation in oligotrophic liquid medium: at room temperature, strain SCSIO 41031 was inoculated into one hundred twenty 1-L conical flasks, each containing soluble starch 2.5 g, tryptone 0.25 g, sea salt 5 g, and 250 mL water. Fermentation proceeded for 30 days. The fermentation material was extracted three times with ethyl acetate to give a brown extract (45.8 g).

### 3.4. Isolation and Purification

The EtOAc extract from solid rice medium fermentation was subjected to vacuum liquid chromatography (VLC) on a silica gel column, eluted with a stepwise gradient of CH_2_Cl_2_-MeOH (99:1–0:100, *v*/*v*) to afford eleven fractions based on TLC analysis. Fr.2 was subdivided into nine subfractions (Frs.2-1–2-9) by ODS silica gel chromatography eluted with MeOH/H_2_O (10–100%). Fr.2-3 was further purified by semi-preparative HPLC (45% MeOH/H_2_O, 2 mL/min) to yield **1** (6.98 mg, t_R_ 22 min) and **5** (11.23 mg, t_R_ 27 min). Fr.2-5 was further purified by HPLC (60% MeOH/H_2_O, 2 mL/min) to yield **6** (28.34 mg, t_R_ 22 min) and **7** (23.56 mg, t_R_ 28 min). Fr.2-6 was further purified by HPLC (65% MeOH/H_2_O, 2 mL/min) to afford **8** (27.12 mg, t_R_ 25 min), **9** (22.11 mg, t_R_ 27 min) and **10** (16.75 mg, t_R_ 31 min). Additionally, Fr.3 was subdivided into seven subfractions (Frs. 3-1–3-7) by ODS silica gel chromatography eluted with MeOH/H_2_O (10-100%). Fr.3-5 was separated by semi-preparative HPLC (25% MeOH/H_2_O, 2 mL/min) to afford **2** (34.45 mg, t_R_ 31 min) and **3** (19.88 mg, t_R_ 39 min). Fr.4 was subdivided into nine subfractions (Frs.4-1–4-9) by ODS silica gel chromatography eluted with MeOH/H_2_O (10–100%). Fr.4-7 was further purified by semi-preparative HPLC (75% MeOH/H_2_O, 2 mL/min) to yield **4** (18.17 mg, t_R_ 19 min) and **11** (20.06 mg, t_R_ 28 min). Fr.5 was subdivided into ten subfractions (Frs.5-1–5-10) by Sephadex LH-20 column chromatography with CH_2_Cl_2_/MeOH (20–100%). Fr.5-9 was further purified by semi-preparative HPLC (85% MeOH/H_2_O, 2 mL/min) to yield **12** (13.45 mg, t_R_ 25 min), **13** (17.66 mg, t_R_ 31 min) and **14** (19.34 mg, t_R_ 35 min). Fr.6 was subdivided into five subfractions (Frs.6-1–6-5) by Sephadex LH-20 column chromatography with CH_2_Cl_2_/MeOH (30–100%). Fr.6-2 was further purified by semi-preparative HPLC (60% MeOH/H_2_O, 2 mL/min) to yield **16** (23.22 mg, t_R_ 21 min). Fr.7 was subdivided into eight subfractions (Frs.7-1–7-8) by ODS silica gel chromatography eluted with MeOH/H_2_O (30–100%). Fr.7-6 was further purified by semi-preparative HPLC (60% MeOH/H_2_O, 2 mL/min) to yield **15** (16.54 mg, t_R_ 37 min).

The EtOAc extract from high-salinity solid rice medium fermentation was subjected to VLC on a silica gel column, eluted with a stepwise gradient of CH_2_Cl_2_-MeOH (99:1–0:100, *v*/*v*) to afford six fractions based on TLC analysis. Fr.4 was subdivided into seven subfractions (Frs.4-1–4-7) by ODS silica gel chromatography eluted with MeOH/H_2_O (30–100%). Fr.4-4 was further purified by semi-preparative HPLC (55% MeOH/H_2_O, 2 mL/min) to yield **19** (23.67 mg, t_R_ 15 min). Fr.5 was subdivided into eight subfractions (Frs.5-1–5-8) by ODS silica gel chromatography eluted with MeOH/H_2_O (30–100%). Fr.5-5 was further purified by semi-preparative HPLC (60% MeOH/H_2_O, 2 mL/min) to yield **15** (15.44 mg, t_R_ 26 min), **16** (13.26 mg, t_R_ 18 min) and **17** (5.67 mg, t_R_ 35 min). Fr.6 was subdivided into six subfractions (Frs.6-1–6-6) by ODS silica gel chromatography eluted with MeOH/H_2_O (40–100%). Fr.6-8 was further purified by semi-preparative HPLC (80% MeOH/H_2_O, 2 mL/min) to yield **18** (17,13 mg, t_R_ 21.1 min).

The EtOAc extract from oligotrophic liquid medium fermentation was subjected to VLC on a silica gel column, eluted with a stepwise gradient of CH_2_Cl_2_-MeOH (99:1–0:100, *v*/*v*) to afford six fractions based on TLC analysis. Fr.4 was subdivided into six subfractions (Frs.4-1–4-6) by ODS silica gel chromatography eluted with MeOH/H_2_O (20–100%). Fr.4-7 was further purified by semi-preparative HPLC (35% MeOH/H_2_O, 2 mL/min) to yield **20** (9.88 mg, t_R_ 31 min) and **21** (12.34 mg, t_R_ 37 min). Fr.5 was subdivided into five subfractions (Frs.5-1–5-5) by ODS silica gel chromatography eluted with MeOH/H_2_O (20–100%). Fr.5-3 was further purified by semi-preparative HPLC (30% MeOH/H_2_O, 2 mL/min) to yield **22** (19.45 mg, t_R_ 21 min).

6,6′-Diacetyl-1,1′-dihydroxy-3,3′-dimethoxydibenzyl ether (**1**): pink solid; ^1^H NMR (CD_3_OD, 500 MHz) and ^13^C NMR (CD_3_OD, 125 MHz), Table 1; HRESIMS *m*/*z* 375.1433 [M + H]^+^ (calculated for C_20_H_23_O_7_, 375.1438), 397.1263 [M + Na]^+^ (calculated for C_20_H_22_NaO_7_, 397.1258).

Esterwortmannolol (**17**): white solid; ^1^H NMR (DMSO-*d*_6_, 500 MHz) and ^13^C NMR (DMSO-*d*_6_, 125 MHz), Table 2; HRESIMS *m*/*z* 407.1469 [M + Na]^+^ (calculated for C_22_H_2_4NaO_6_, 407.1465), 385.1645 [M + H]^+^ (calculated for C_22_H_25_O_6_, 385.1646).

Pestalpolyol I (**20**): white solid; ^1^H NMR (DMSO-*d*_6_, 500 MHz) and ^13^C NMR (DMSO-*d*_6_, 125 MHz), Table 3; HRESIMS *m*/*z* 401.2662 [M + Na]^+^ (calculated for C_23_H_38_NaO_4_, 401.2662).

6,6′-Diacetyl-1,1′-dihydroxy-3,3′-dimethoxydibenzyl ether (**1**) obtained as colorless crystals through the process of slow evaporation at room temperature in a mixture of MeOH and H_2_O (1:1). The crystal’s information was collected using Cu Kα radiation on an XtalLAB PRO single-crystal diffractometer. The X-ray crystal structure of **1** was determined using SHELXS97, expanded by difference Fourier techniques, and refined through full-matrix least-square calculation. Crystallographic data (excluding structure factors) for structure **1** in this paper were deposited with the Cambridge Crystallographic Data Centre as supplementary publication number CCDC 2527973. Copies of the data can be obtained, free of charge, on application to CCDC, 12 Union Road, Cambridge CB21EZ, UK (fax: +44 (0)1223 336408).

### 3.5. ECD Calculation Method

The general procedure for ECD calculations is generally divided into four main steps: conformational optimization, conformational analysis, spectrum calculation, and spectral fitting. Conformational optimization was carried out using ChemBio3D 19.0 software with the MMFF94 force field. Conformational analysis was carried out by combining a molecular mechanics-based search for low-energy conformers with quantum chemical conformational optimization. Initially, Spartan 14 software was used to generate and screen conformations, only conformers with populations exceeding 5% were retained as dominant conformations. Subsequently, density functional theory (DFT) calculations were performed using Gaussian 06 software at the B3LYP/6-31G(d) level to further optimize the low-energy conformers and confirm the dominant ones. For each dominant conformation, UV/ECD spectra were calculated in methanol solvent using DFT at the B3LYP/6-31+G(d,p) level. Solvent effects were accounted for using the SCRF-PCM method. The resulting ECD spectra for individual conformers were then Boltzmann-weighted averaged according to their thermodynamic distributions, yielding the final calculated ECD spectrum for the target compound. A wavelength correction factor (i.e., a shift value) was determined by comparing the calculated UV spectrum with the experimental UV spectrum of the compound. This correction factor was applied to adjust the calculated ECD spectrum accordingly. Finally, the corrected ECD spectrum was compared with the experimental ECD spectrum to determine the absolute configuration of the compound.

### 3.6. Bioactivity Assay

Cell viability of tumor cell lines (Hela, HL-60, K562, Molt-4, ACHN, 786-O, and OS-RC-2) was evaluated by the CCK-8 assay. All cell lines were purchased from the Shanghai Cell Bank, Chinese Academy of Sciences. Detailed procedures were performed according to reference [30].

The protective effect against Aβ_25–35_-induced apoptosis in primary cortical neurons was evaluated by the MTT assay, and the detailed procedure was performed according to the method described in reference [31].

Anti-inflammatory activity was assessed by measuring nitric oxide (NO) production using the Griess reaction. Mouse RAW264.7 macrophages were cultured in vitro and stimulated with lipopolysaccharide (LPS) in the presence of test compounds at a final concentration of 10 μM. After incubation, culture supernatants were collected and NO levels were determined spectrophotometrically. The experimental protocol followed the procedure reported in reference [32].

## 4. Conclusions

In conclusion, this study applied the OSMAC strategy to *Aspergillus sclerotiorum* SCSIO 41031. Large-scale fermentation was performed using three optimized culture media. Chromatographic separation and spectroscopic analysis yielded a total of 22 compounds, including 3 new compounds and 19 known compounds. In our extensive activity screening, multiple biological effects were observed. Notably, compounds **12**, **13**, and **22** exhibited cytotoxicity. While compounds **1**, **5**, **6**, **8**, **15**, and **16** provided weak protection to primary cortical neurons, compounds **5**, **6**, and **13** showed weak anti-inflammatory activity. In contrast, compounds **7**, **9**, and **15** were found to have weak pro-inflammatory effects. These findings demonstrate the effectiveness of the OSMAC approach in unlocking the chemical diversity of *A. sclerotiorum* SCSIO 41031 and highlight this strain as a promising source of bioactive natural products.

## Figures and Tables

**Table 1:** ^1^H and ^13^C NMR data for **1** (700, 175 MHz, MeOD, TMS, *δ* ppm).

C/H	*δ*_C_	*δ*_H_ (*J* in Hz)	gCOSY	gHMBC
1 1′	163.4	-		
2 2′	116.8	2.58 m		
3 3′	165.5	-		
4 4′	103.5	-	H5	C-2, C-6
5 5′	134.4	6.64 m	H4	C-8
6 6′	115.5	7.9 m		
7 7′	52.9	-		C-1, C-2
8 8′	205	4.7 s		
9 9′	56.5	-		C-3
10 10′	26.4	3.94 s		C-8

**Table 2:** ^1^H and ^13^C NMR data for **17** (700, 175 MHz, MeOD, TMS, *δ* ppm).

C/H	*δ*_C_	*δ*_H_ (*J* in Hz)	gCOSY	gHMBC	C/H	*δ*_C_	*δ*_H_ (*J* in Hz)	gCOSY	gHMBC
1	55.9	3.71, d (3.8)	H2	C-3	12	28.9	2.32, m; 1.76, m	H11	C-9
2	54.8	3.47, dd (3.7, 2.7)	H1	C-3	13	44.6			
3	61.1	5.23, d (2.7)			14	44.1	2.45, m		
4	122.0				15	25.6	2.73, m	H16	
5	143.2				16	33.4	1.97, m; 1.59, m	H15	
6	146.7				17	82.1	4.77, dd (9.3, 7.2)		C-15, C-18
7	176.2				18	12.1	0.89, s		
8	135.9				19	29.4	1.70, s		
9	161.7				20	173.0			
10	42.5				21	20.9	2.08, s		C-20
11	25.6	2.80, m; 2.72, m	H12		22	147.1	7.82, s		C-3

**Table 3:** ^1^H and ^13^C NMR data for **20** (500, 125 MHz, DMSO-*d*_6_, TMS, *δ* ppm).

C/H	*δ*_C_	*δ*_H_ (*J* in Hz)	gCOSY	gHMBC	C/H	*δ*_C_	*δ*_H_ (*J* in Hz)	gCOSY	gHMBC
1	195.5	9.37, s			13	80.8	3.58, dd (7.9, 3.6)	H12	C-11
2	137.9				14	137.5			
3	159.3	6.59, dd (9.7, 1.6)	H4		15	119.8	5.35, d (6.7)	H16	C-13, C-23
4	37.2	2.79, m	H3, H18	C-3	16	12.8	1.54, m; 1.57, m	H15	C-14
5	80.2	3.77, dd (7.8, 3.9)		C-6, C-19	17	9.2	1.67, d (1.3)		C-2, C-3
6	135.5				18	16.5	0.87, d (6.8)	H4	C-4
7	131.1	5.26, d (9.2)	H8	C-5	19	11.6	1.54, m; 1.57, m		
8	35.9	2.46, q (7.7)	H7, H9, H20		20	17.7	0.73, d (5.7)	H8	C-7, C-8
9	80.8	3.58, dd (7.9, 3.6)	H8		21	11.5	1.54, m; 1.57, m		C-9, C-10
10	136.1				22	17.5	0.73, d (5.7)	H12	C-11, C-12
11	130.3	5.19, d (9.1)	H12	C-9	23	11.1	1.57, m		C-13
12	35.6	2.46, q (7.7)	H11, H13, H22						

**Table 4:** Cytotoxic of compounds **12**, **13** and **22**.

Cell Lines	IC_50_ (μM)			
	12	13	22	Positive Control ^a^
K562	4.76	4.33	>100	0.16
Molt-4	2.11	2.39	>100	0.03
Hela	6.35	6.12	50.02	0.10
HL60	2.34	2.44	>100	0.03

^a^ Trichostatin A was used as a positive control in the cytotoxicity bioassay of seven human cancer cell lines.

## Data Availability

The original contributions presented in this study are included in the article and Supplementary Materials. Further inquiries can be directed to the corresponding authors.

## Supplementary Materials

The following supporting information can be downloaded at: https://www.mdpi.com/article/10.3390/md24040128/s1, Figures S1–S21: ^1^H NMR, ^13^C NMR, DEPT, HSQC, HMBC, COSY, NOESY and HRESIMS of compounds **1**, **17** and **20**. Table S1: Energies of all calculated conformers of (1*R*, 2*S*, 3*R*, 10*R*, 13*S*, 14*R*, 17*S*)-**1**. Table S2: Cartesian coordinates of all conformers of (1*R*, 2*S*, 3*R*, 10*R*, 13*S*, 14*R*, 17*S*)-**1**. Tables S3–S21: ^1^H NMR and ^13^C NMR data of compounds **2**–**16**, **18**–**19**, **21**–**22** and comparison with literature values [11,12,13,14,15,16,17,18,19,20,21,22,23,24,25,26,27]. Figure S22: Cytotoxicity of compounds **12** and **13** against K562, Molt-4, HL60 and Hela cell lines.
