# Supplementary material for: Chemical Diversity and Antitumor Metabolites from Soft Coral-Derived Fungus Aspergillus sclerotiorum SCSIO 41031 via OSMAC Strategy
Source: Mar Drugs. 2026 Mar 31;24(4):128. doi: 10.3390/md24040128 (PMC13118068; doi:10.3390/md24040128)
Supplement: Supplementary file 1 [file marinedrugs-24-00128-s001.zip › marinedrugs-4163491-supplementary.pdf]

Chemical Diversity and Antitumor Metabolites from Soft Coral-Derived Fungus *Aspergillus sclerotiorum* SCSIO 41031 via OSMAC Strategy

Juan Gao [1,2], Jieyi Long [1,3], Xiaoyan Pang [1], Xuefeng Zhou [1], Yonghong Liu [1,2,*], and Bin Yang [1,*]

[1] Guangdong Key Laboratory of Marine Materia Medica/State Key Laboratory of Tropical Oceanography, South China Sea Institute of Oceanology, Chinese Academy of Sciences, Guangzhou 510301, China; gaojuan24@mails.ucas.ac.cn (J.G.); xypang@scsio.ac.cn (X.P.); xfzhou@scsio.ac.cn (X.Z.)

[2] University of Chinese Academy of Sciences, 19 Yuquan Road, Beijing 100049, P. R. China

[3] Infinitus (China) Company Ltd., Guangzhou 510623, China; Anna.Long@infinitus-int.com

[*] Correspondence: yonghongliu@scsio.ac.cn (Y.L.); yangbin@scsio.ac.cn (B.Y.)

1. NMR, MS spectra of new compounds 1, **17** and **20** **4**
Figure S1. ^{1}H NMR spectrum of **1** (DMSO-*d*$_6$, 700 MHz) 4
Figure S2. ^{13}C NMR spectrum of **1** (CD_3OD-*d*$_6$, 175 MHz) 4
Figure S3. DEPT spectrum of **1** (CD_3OD-*d*$_6$) 5
Figure S4. HSQC spectrum of **1** (CD_3OD-*d*$_6$) 5
Figure S5. ^{1}H-^{1}H COSY spectrum of **1** (CD_3OD-*d*$_6$) 6
Figure S6. HMBC spectrum of **1** (CD_3OD-*d*$_6$) 6
Figure S7. HRESIMS spectrum of **1** 7
Figure S8. ^{1}H NMR spectrum of **17** (CD_3OD-*d*$_6$, 700 MHz) 7
Figure S9. ^{13}C NMR spectrum of **17** (CD_3OD-*d*$_6$, 175 MHz) 8
Figure S10. HSQC spectrum of **17** (CD_3OD-*d*$_6$) 8
Figure S11. ^{1}H-^{1}H COSY spectrum of **17** (CD_3OD-*d*$_6$) 9
Figure S12. HMBC spectrum of **17** (CD_3OD-*d*$_6$) 9
Figure S13. NOESY spectrum of **17** (CD_3OD-*d*$_6$) 10
Figure S14. HRESIMS spectrum of **17** 10
Figure S15. ^{1}H NMR spectrum of **20** (DMSO-*d*$_6$, 500 MHz) 11
Figure S16. ^{13}C NMR spectrum of **20** (DMSO-*d*$_6$, 125 MHz) 11
Figure S17. HSQC spectrum of **20** (DMSO-*d*$_6$) 12
Figure S18. ^{1}H-^{1}H COSY spectrum of **20** (DMSO-*d*$_6$) 12
Figure S19. HMBC spectrum of **20** (DMSO-*d*$_6$) 13
Figure S20. NOESY spectrum of **20** (DMSO-*d*$_6$) 13
Figure S21. HRESIMS spectrum of **20** 14
2. ECD calculations **14**
Table S1. Energies of all calculated conformers of (1*R*, 2*S*, 3*R*, 10*R*, 13*S*, 14*R*, 17*S*)-**1** 14
Table S2. Cartesian coordinates of all conformers of (1*R*, 2*S*, 3*R*, 10*R*, 13*S*, 14*R*, 17*S*)-**1** 15
3. The physicochemical data of the known compounds 2-16, **18-19** and **21-22** **20**
Table S3. ^{1}H NMR and ^{13}C NMR Data of Compound **2** and Comparison with Literature Values 20
Table S4. ^{1}H NMR and ^{13}C NMR Data of Compound **3** and Comparison with Literature Values 21
Table S5. ^{1}H NMR and ^{13}C NMR Data of Compound **4** and Comparison with Literature Values 22

Table S6. ^{1}H NMR and ^{13}C NMR Data of Compound **5** and Comparison with Literature Values 22
Table S7. ^{1}H NMR and ^{13}C NMR Data of Compound **6** and Comparison with Literature Values 23
Table S8. ^{1}H NMR and ^{13}C NMR Data of Compound **7** and Comparison with Literature Values 23
Table S9. ^{1}H NMR and ^{13}C NMR Data of Compound **8** and Comparison with Literature Values 24
Table S10. ^{1}H NMR and ^{13}C NMR Data of Compound **9** and Comparison with Literature Values 24
Table S11. ^{1}H NMR and ^{13}C NMR Data of Compound **10** and Comparison with Literature Values 25
Table S12. ^{1}H NMR and ^{13}C NMR Data of Compound **11** and Comparison with Literature Values 25
Table S13. ^{1}H NMR and ^{13}C NMR Data of Compound **12** and Comparison with Literature Values 26
Table S14. ^{1}H NMR and ^{13}C NMR Data of Compound **13** and Comparison with Literature Values 26
Table S15. ^{1}H NMR and ^{13}C NMR Data of Compound **14** and Comparison with Literature Values 27
Table S16. ^{1}H NMR and ^{13}C NMR Data of Compound **15** and Comparison with Literature Values 28
Table S17. ^{1}H NMR and ^{13}C NMR Data of Compound **16** and Comparison with Literature Values 29
Table S18. ^{1}H NMR and ^{13}C NMR Data of Compound **18** and Comparison with Literature Values 29
Table S19. ^{1}H NMR and ^{13}C NMR Data of Compound **19** and Comparison with Literature Values 30
Table S20. ^{1}H NMR and ^{13}C NMR Data of Compound **21** and Comparison with Literature Values 31
Table S21. ^{1}H NMR and ^{13}C NMR Data of Compound **22** and Comparison with Literature Values 31
4. Biological evaluation 33
Figure S22. Cytotoxicity of compounds **12** and **13** against K562, Molt-4, HL60 and Hela cell lines 33
References 33

1. NMR, MS spectra of new compounds **1**, **17** and **20**

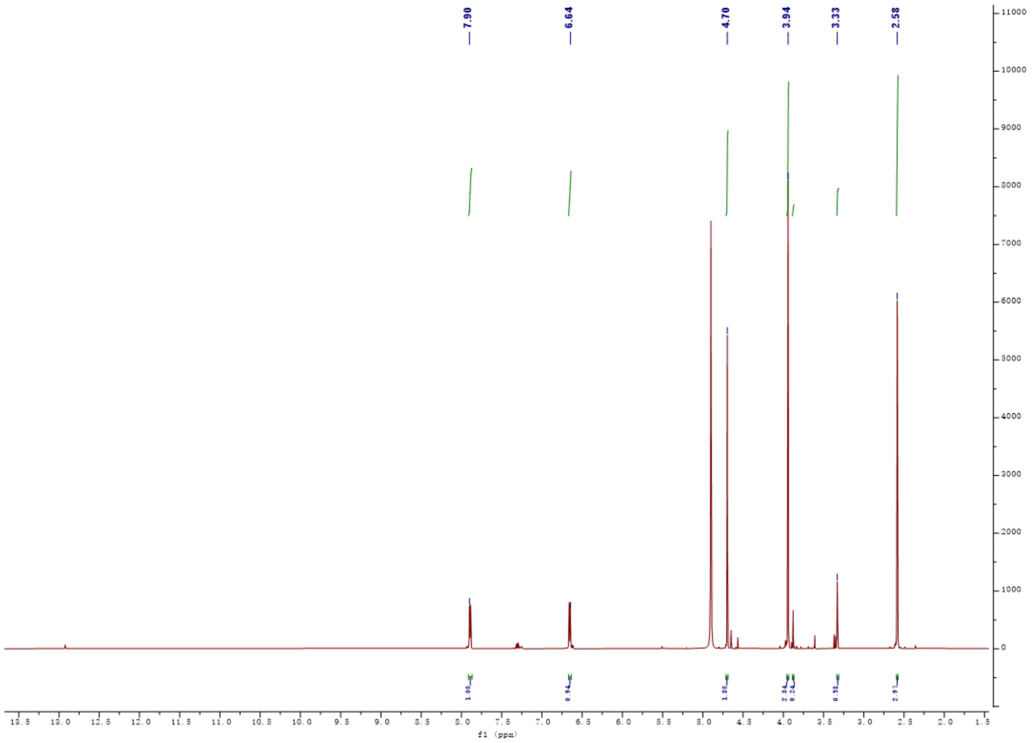

Figure S1. ^{1}H NMR spectrum of **1** (DMSO-d_6, 700 MHz)

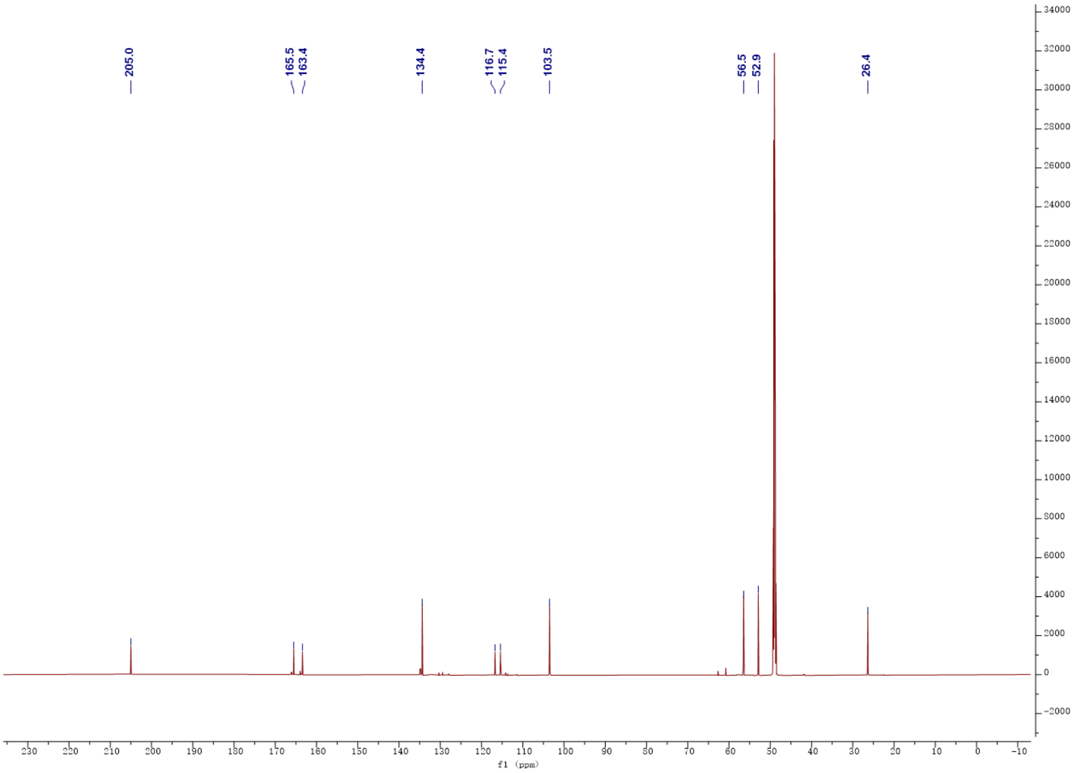

Figure S2. ^{13}C NMR spectrum of **1** (CD_3OD-d_6, 175 MHz)

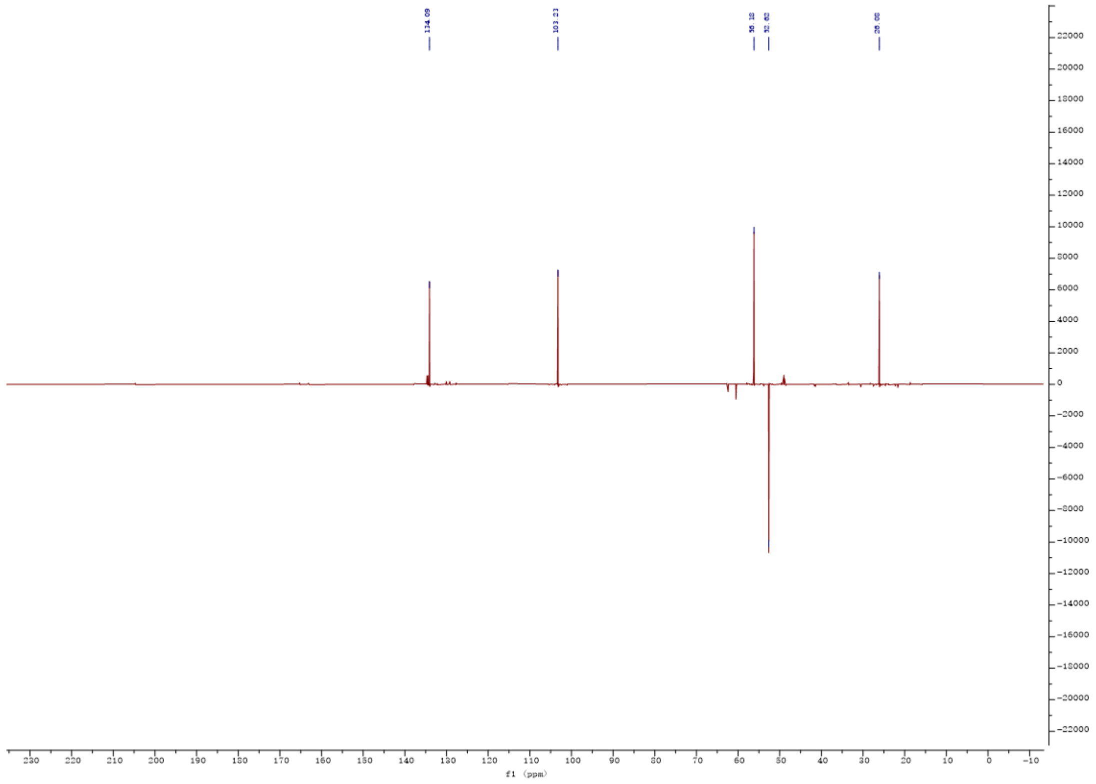

Figure S3. DEPT spectrum of **1** (CD3OD-*d*6)

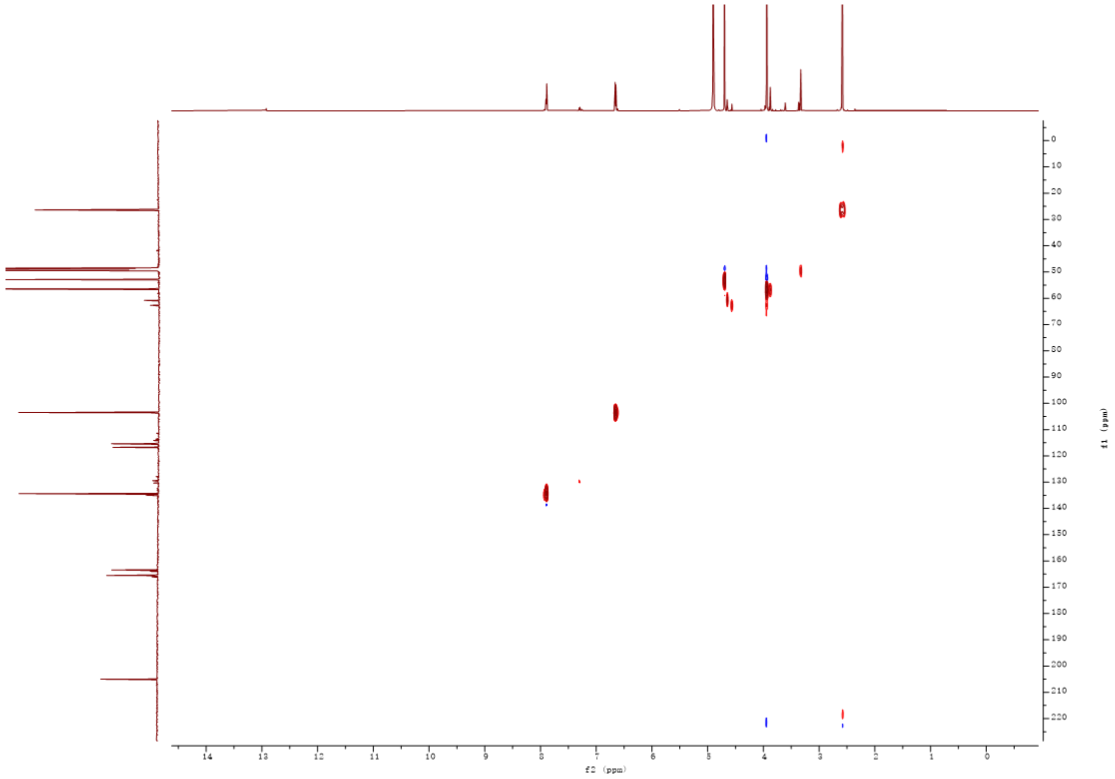

Figure S4. HSQC spectrum of **1** (CD3OD-*d*6)

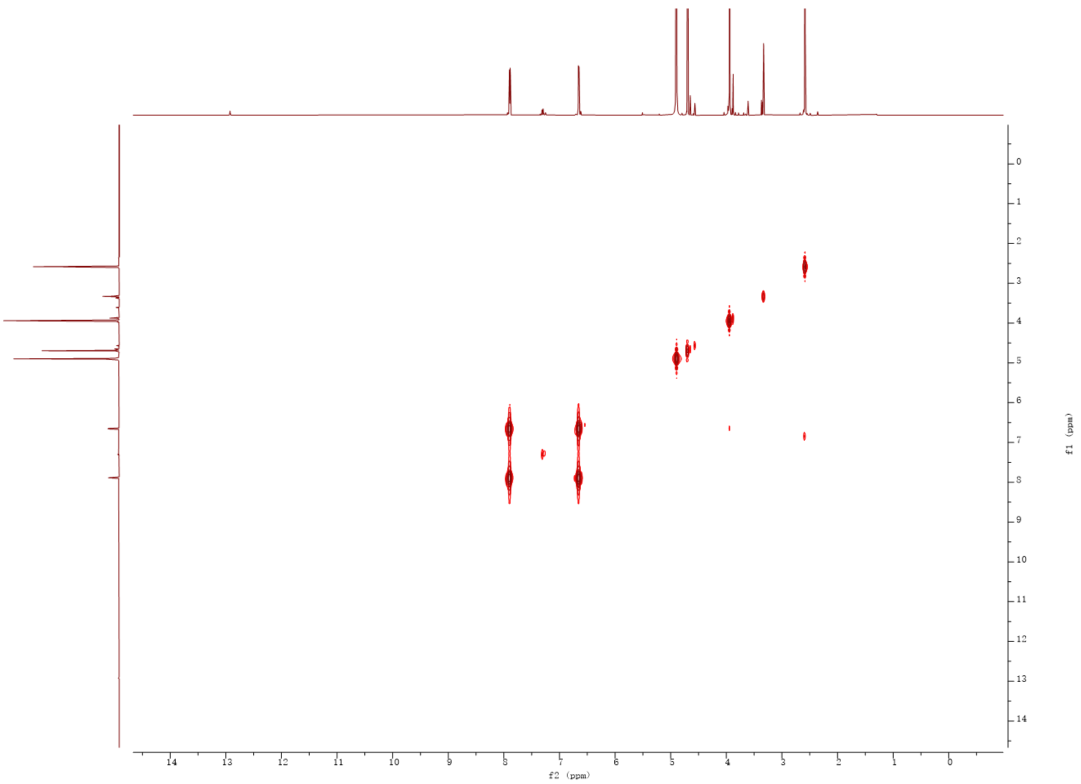

Figure S5. ^{1}H-^{1}H COSY spectrum of **1** (CD_3OD-d_6)

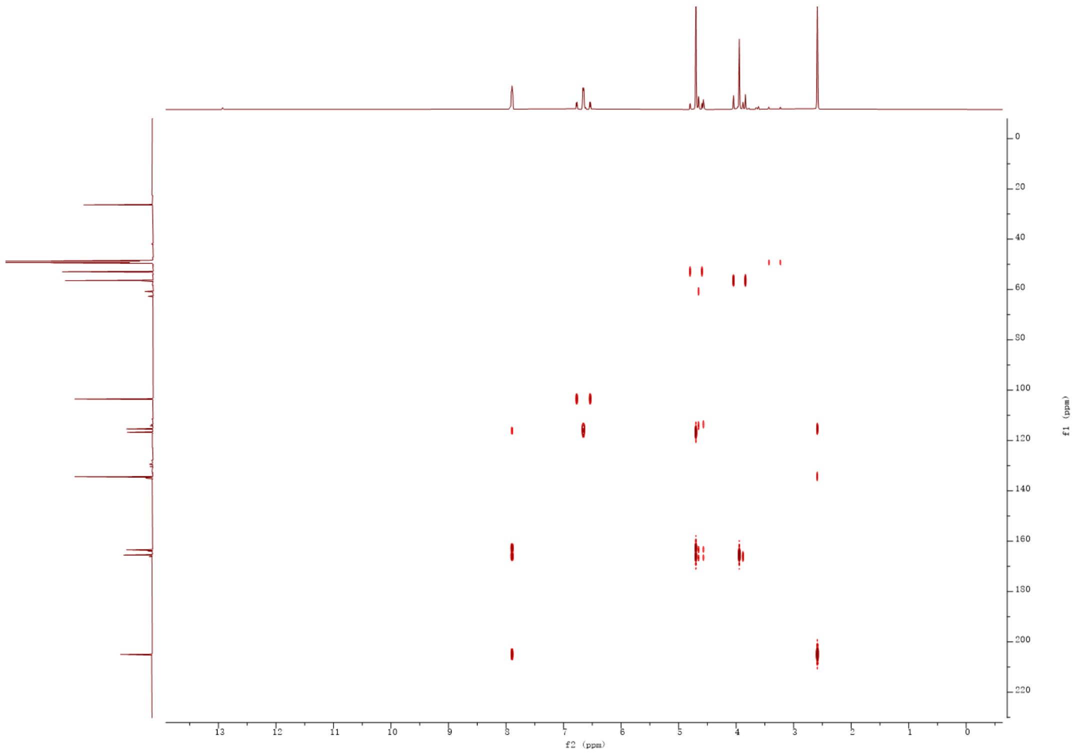

Figure S6. HMBC spectrum of **1** (CD_3OD-d_6)

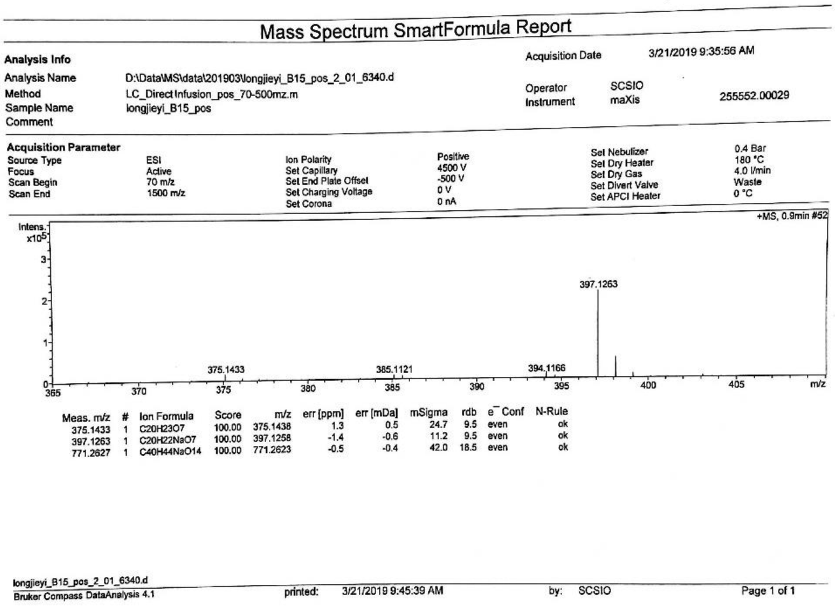

Figure S7. HRESIMS spectrum of **1**

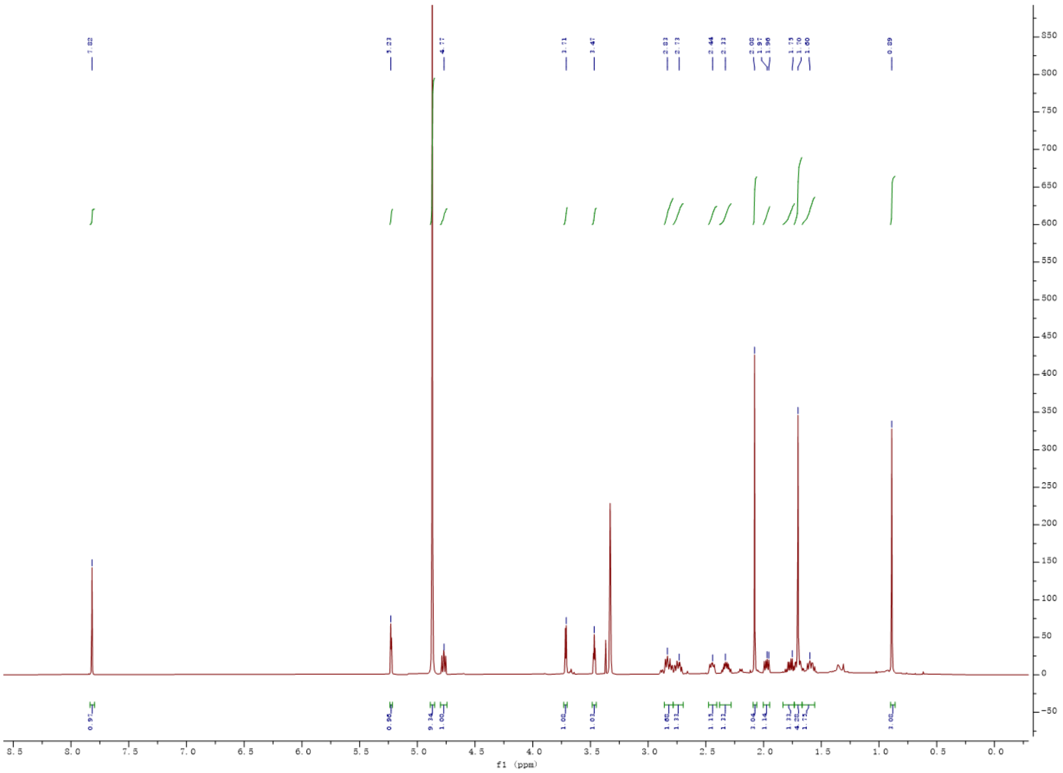

Figure S8. ^{1}H NMR spectrum of **17** (CD_3OD-d_6, 700 MHz)

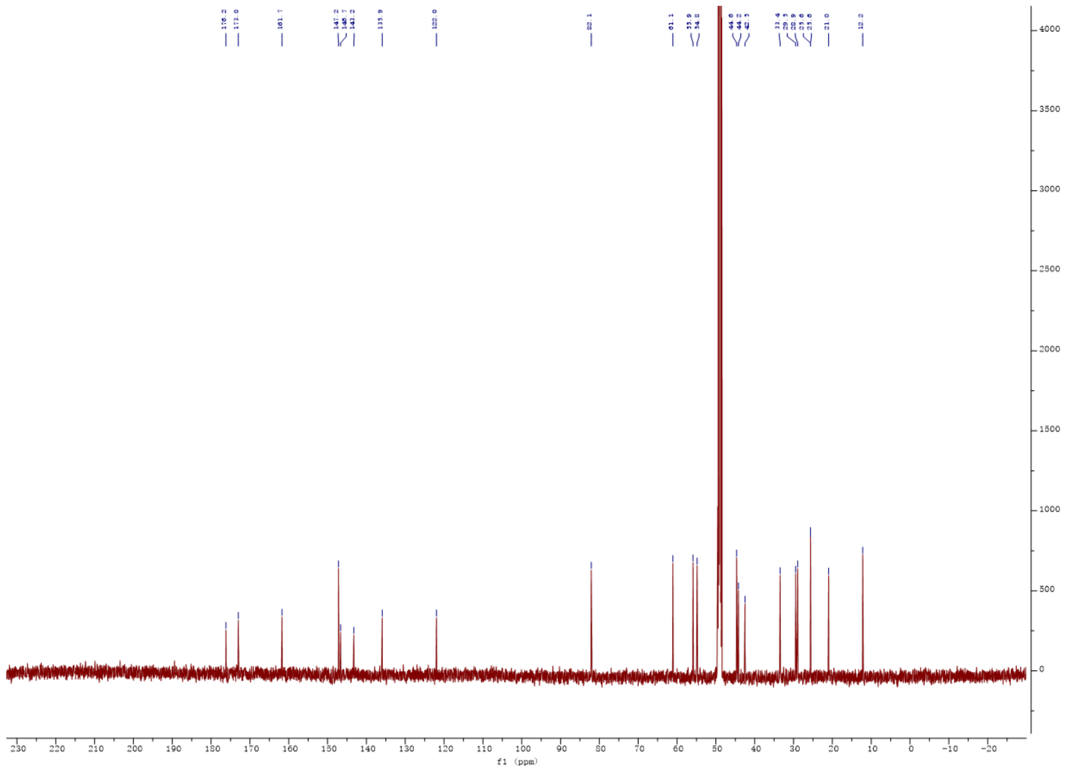

Figure S9. ^{13}C NMR spectrum of **17** (CD_3OD-*d*$_6$, 175 MHz)

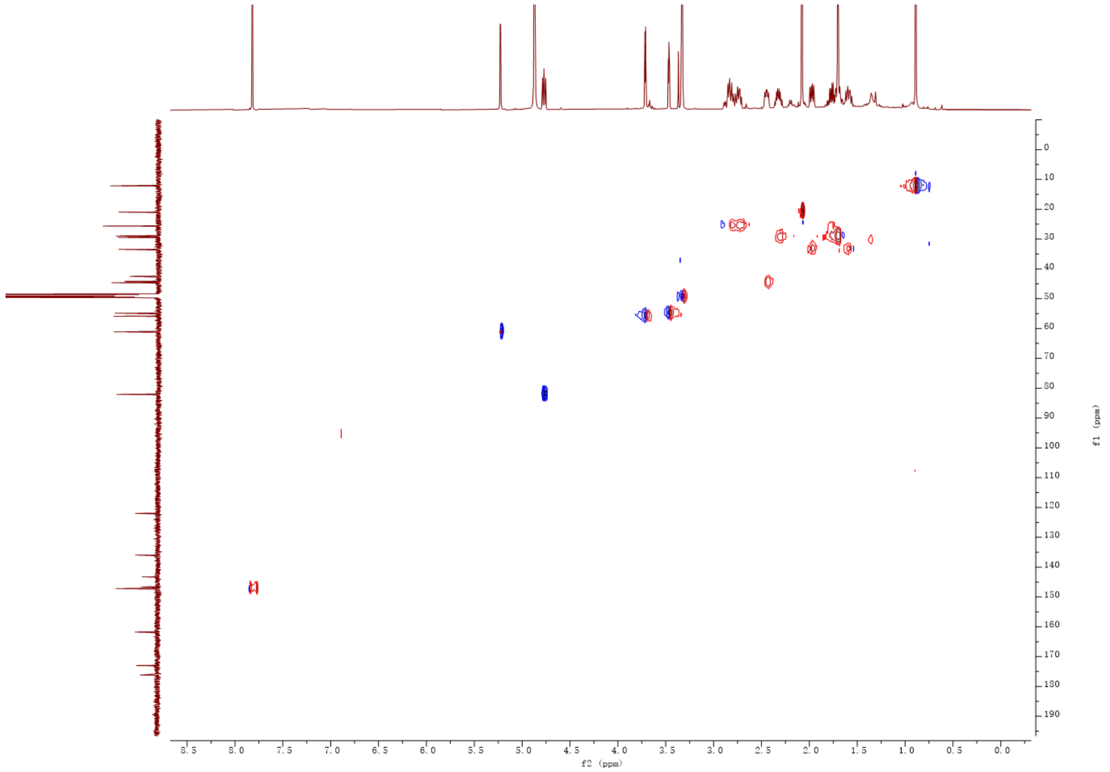

Figure S10. HSQC spectrum of **17** (CD_3OD-*d*$_6$)

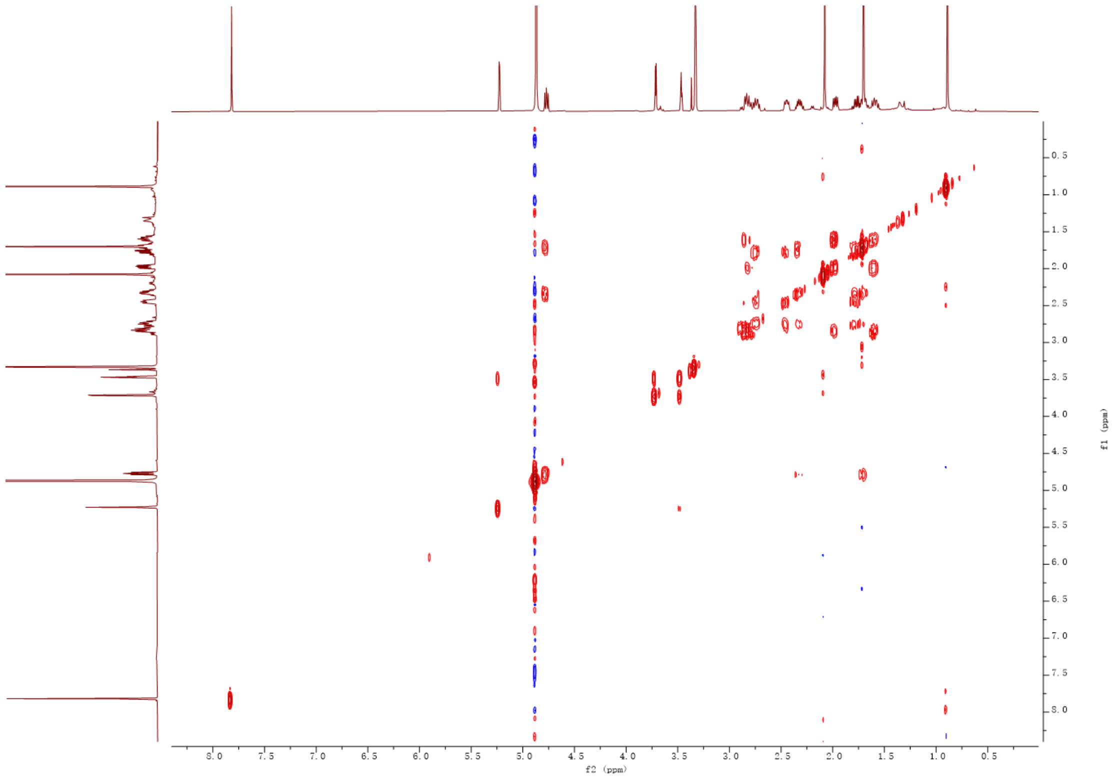

Figure S11. ^{1}H-^{1}H COSY spectrum of **17** (CD_3OD-*d₆*)

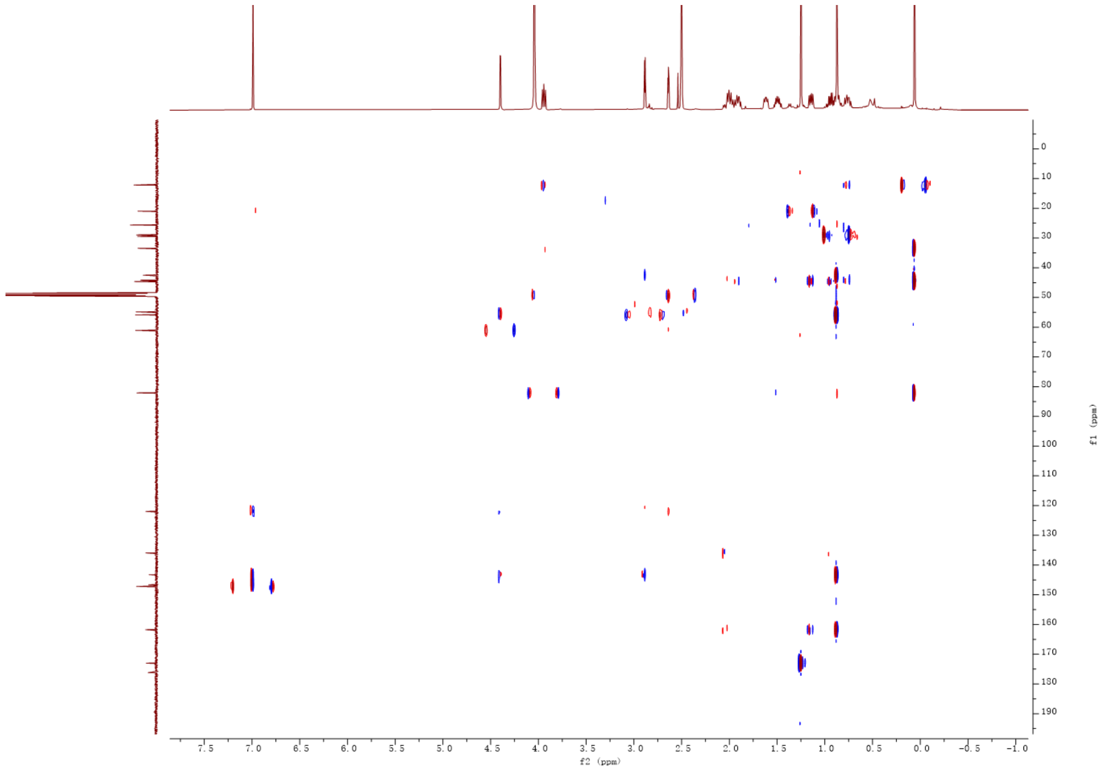

Figure S12. HMBC spectrum of **17** (CD_3OD-*d₆*)

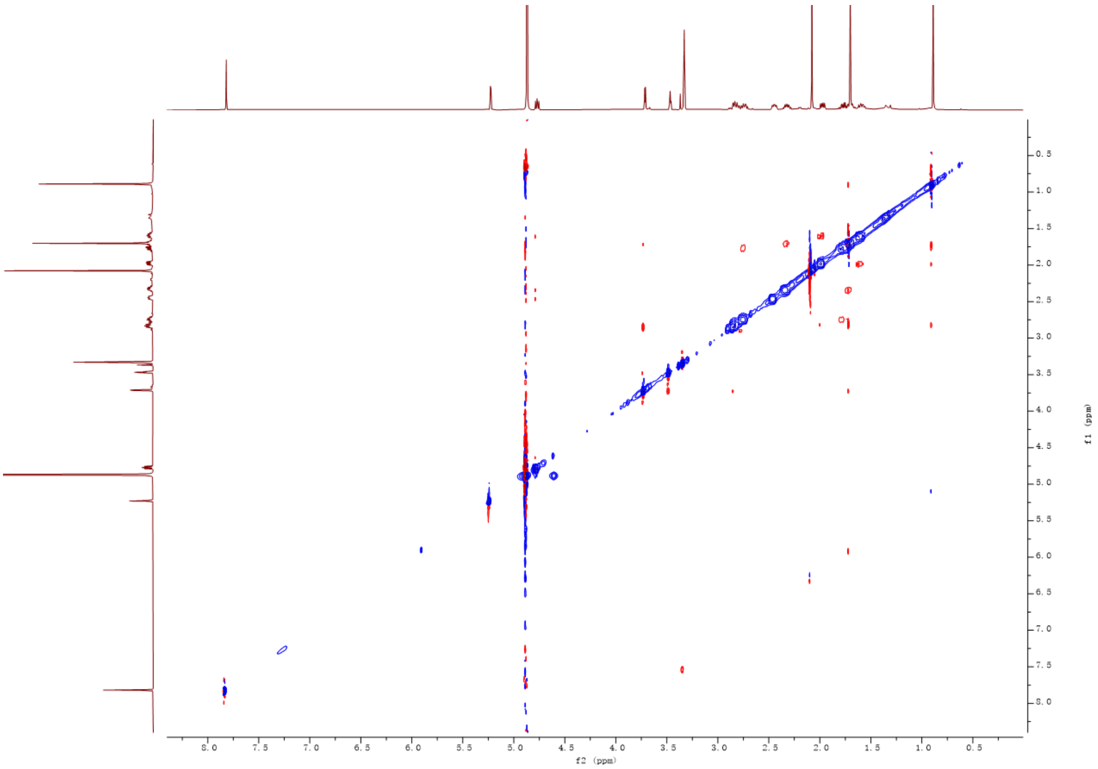

Figure S13. NOESY spectrum of **17** (CD_3OD-d_6)

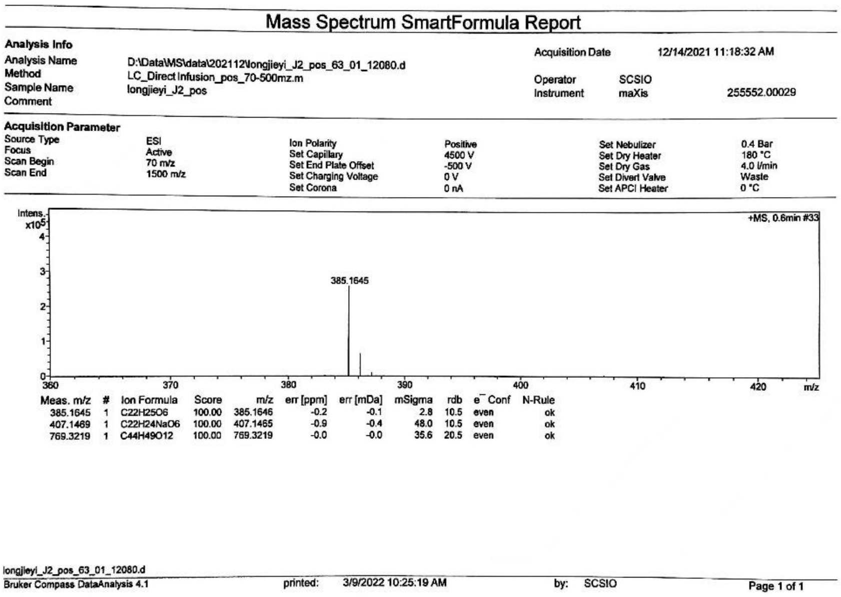

Mass Spectrum SmartFormula Report

Analysis Info

Analysis Name	D:\Data\MS\data\202112\longjieyi_J2_pos_63_01_12080.d	Acquisition Date	12/14/2021 11:18:32 AM
Method	LC_Direct Infusion_pos_70-500mz.m	Operator	SCSIO
Sample Name	longjieyi_J2_pos	Instrument	maXis 255552.00029
Comment			

Acquisition Parameter

Source Type	ESI	Ion Polarity	Positive	Set Nebulizer	0.4 Bar
Focus	Active	Set Capillary	4500 V	Set Dry Heater	180 °C
Scan Begin	70 m/z	Set End Plate Offset	-500 V	Set Dry Gas	4.0 l/min
Scan End	1500 m/z	Set Charging Voltage	0 V	Set Divert Valve	Waste
		Set Corona	0 nA	Set APCI Heater	0 °C

Meas. m/z	#	Ion Formula	Score	m/z	err [ppm]	err [mDa]	mSigma	rdb	e⁻ Conf	N-Rule
385.1645	1	C22H25O6	100.00	385.1646	-0.2	-0.1	2.8	10.5	even	ok
407.1469	1	C22H24NaO6	100.00	407.1465	-0.9	-0.4	48.0	10.5	even	ok
769.3219	1	C44H49O12	100.00	769.3219	-0.0	-0.0	35.6	20.5	even	ok

longjieyi_J2_pos_63_01_12080.d
Bruker Compass DataAnalysis 4.1 printed: 3/9/2022 10:25:19 AM by: SCSIO Page 1 of 1

Figure S14. HRESIMS spectrum of **17**

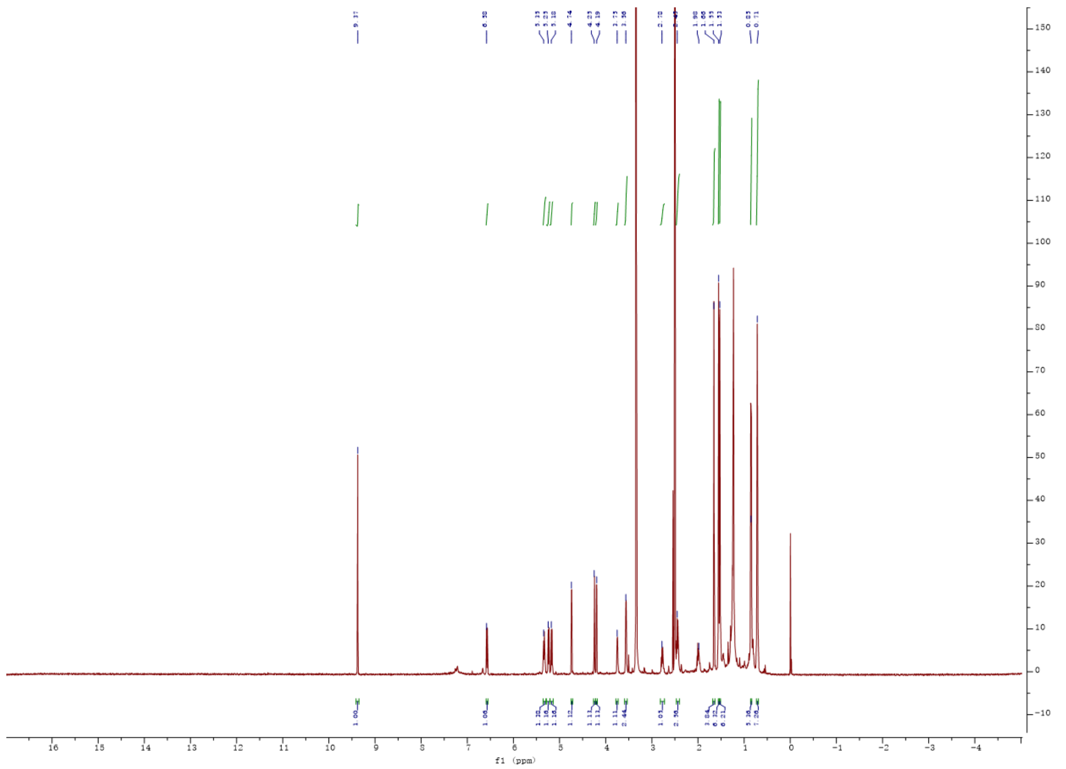

Figure S15. ^{1}H NMR spectrum of **20** (DMSO-d_6, 500 MHz)

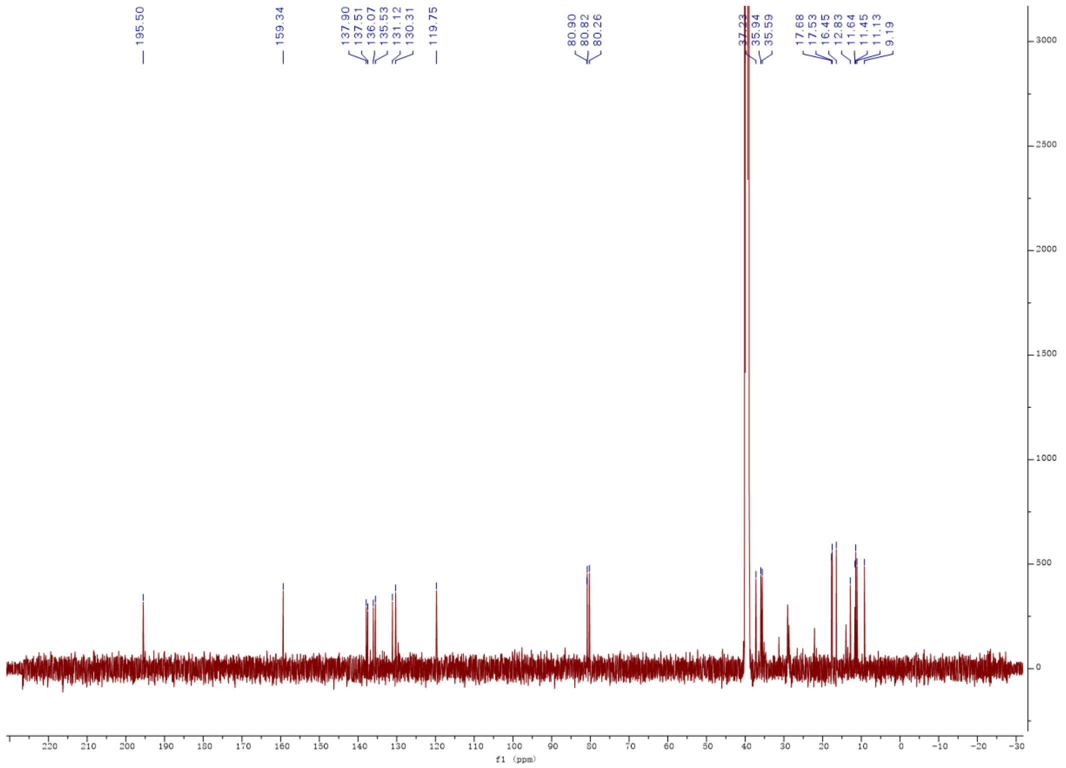

Figure S16. ^{13}C NMR spectrum of **20** (DMSO-d_6, 125 MHz)

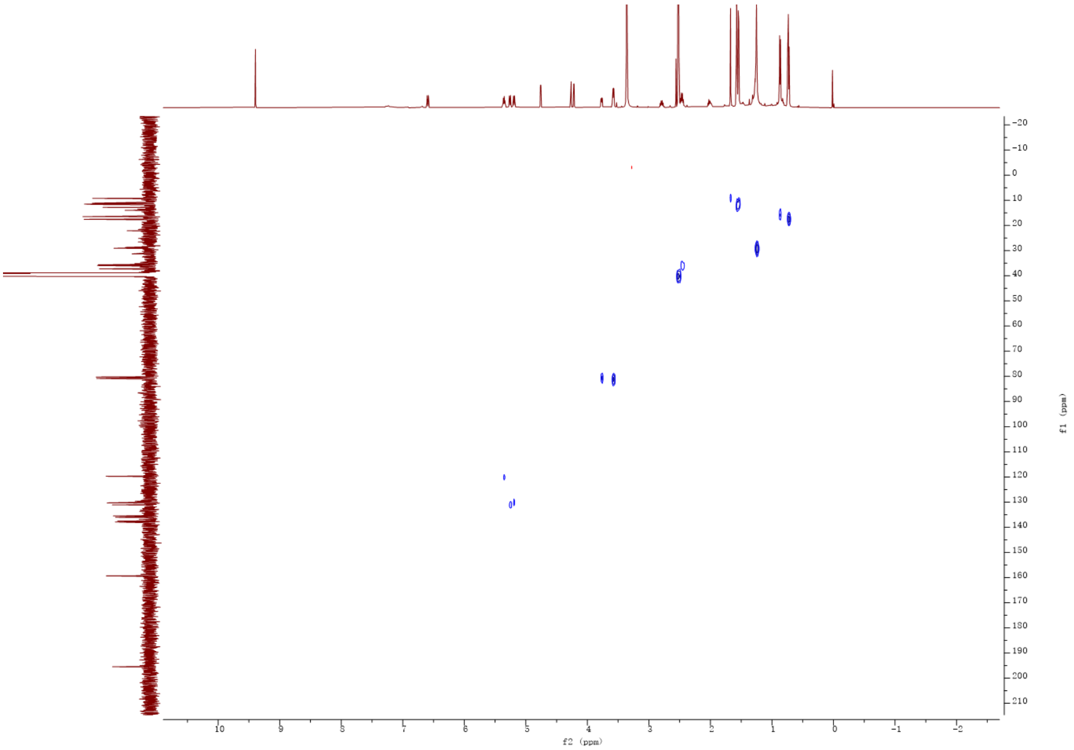

Figure S17. HSQC spectrum of **20** (DMSO-d_6)

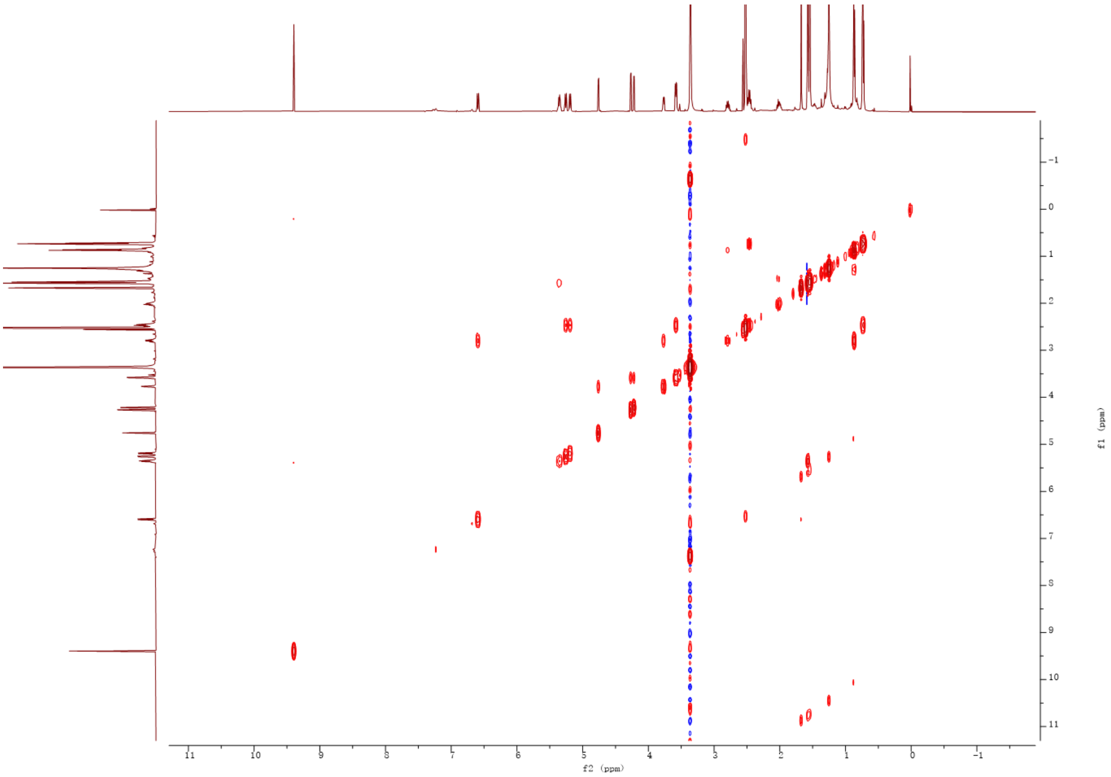

Figure S18. ^{1}H-^{1}H COSY spectrum of **20** (DMSO-d_6)

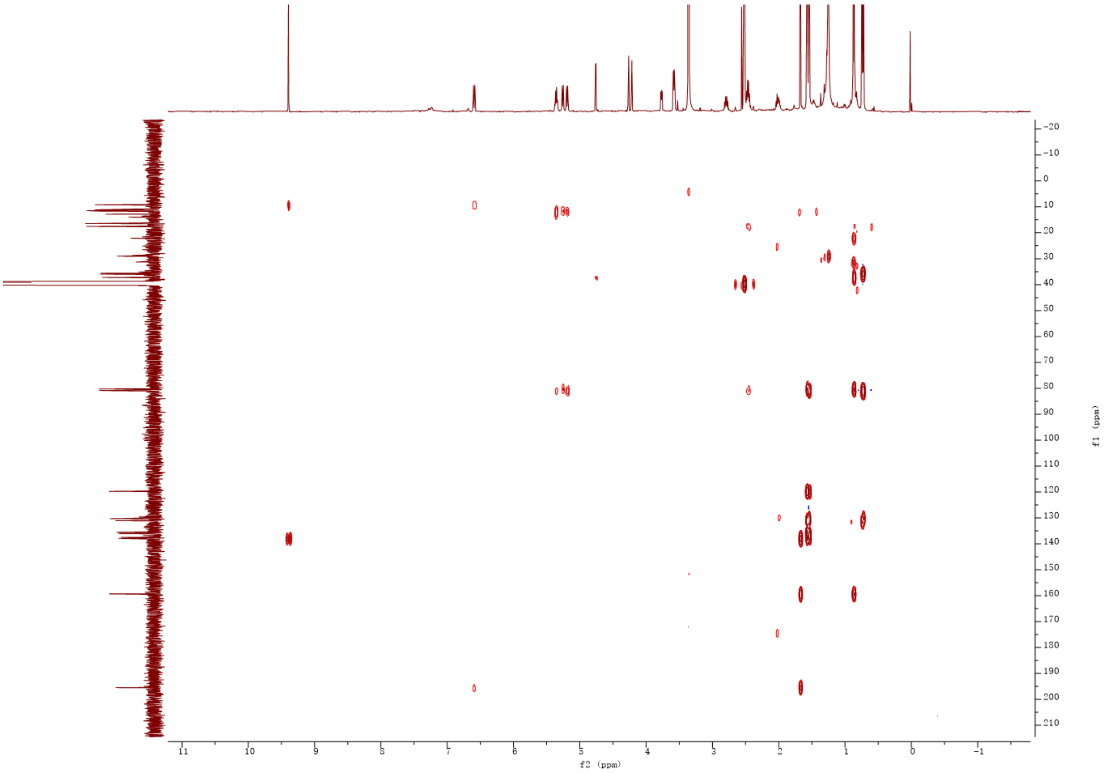

Figure S19. HMBC spectrum of **20** (DMSO-d_6)

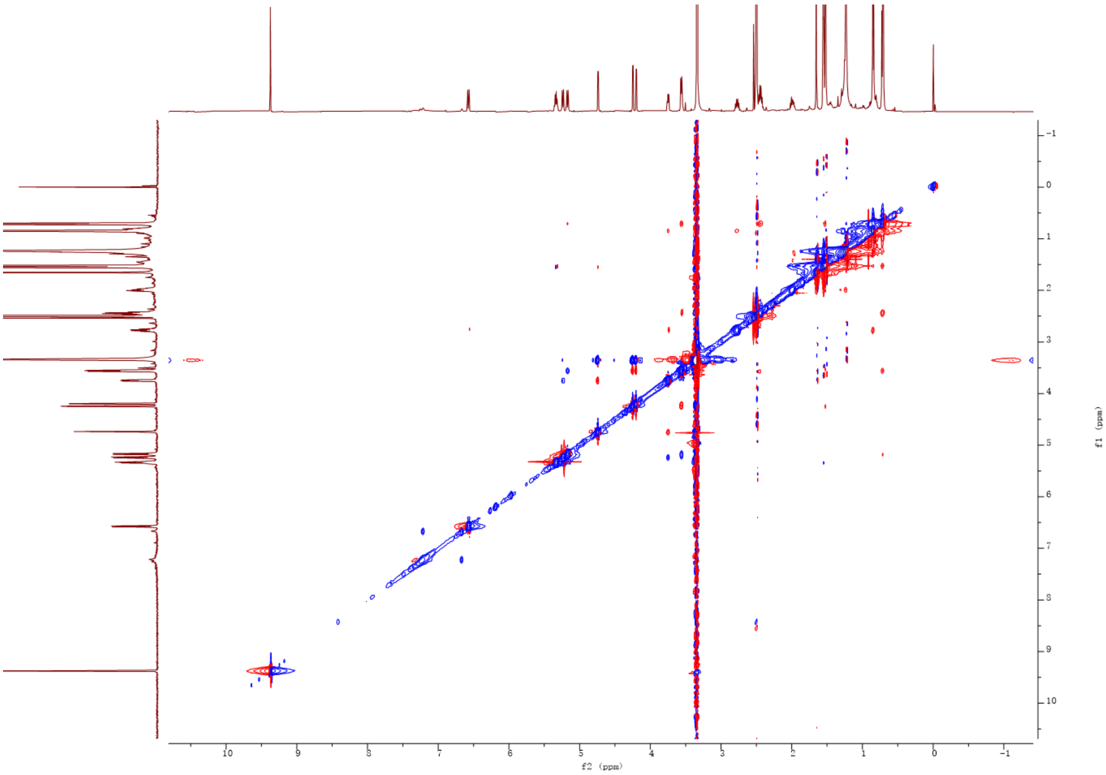

Figure S20. NOESY spectrum of **20** (DMSO-d_6)

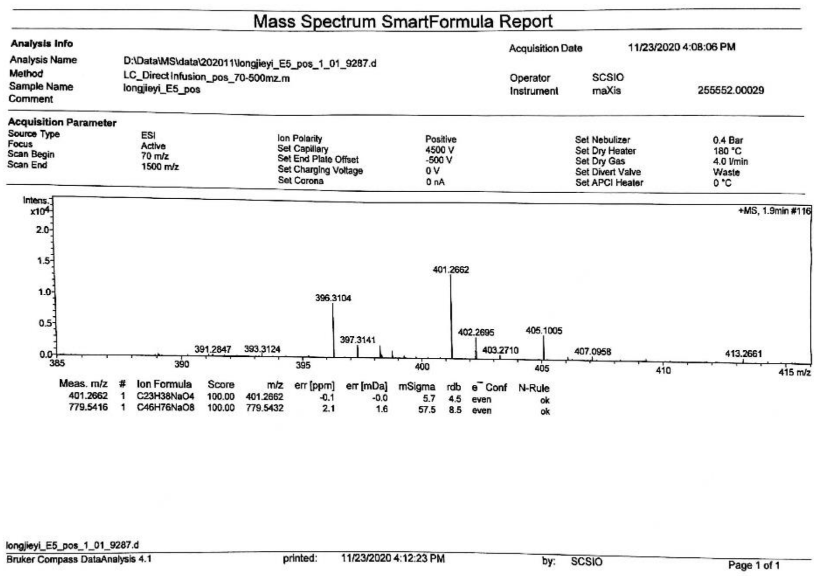

Figure S21. HRESIMS spectrum of **20**

2. ECD calculations

Table S1. Energies of all calculated conformers of (1*R*, 2*S*, 3*R*, 10*R*, 13*S*, 14*R*, 17*S*)-**1**

Configurations	Conformers	E(Hartree)	Energy (kcal/mol)	Population (%)
(1*R*, 2*S*, 3*R*, 10*R*, 13*S*, 14*R*, 17*S*)-**1a**	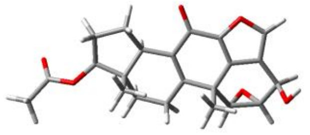	-1304.305412	-818464.037	24.41
(1*R*, 2*S*, 3*R*, 10*R*, 13*S*, 14*R*, 17*S*)-**1b**	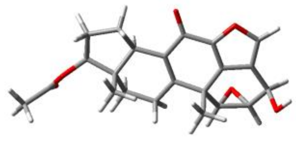	-1304.305274	-818463.9505	21.09
(1*R*, 2*S*, 3*R*, 10*R*, 13*S*, 14*R*, 17*S*)-**1c**	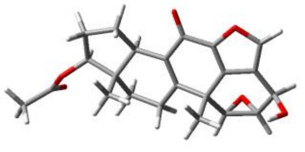	-1304.305388	-818464.0221	23.80
(1*R*, 2*S*, 3*R*, 10*R*, 13*S*, 14*R*, 17*S*)-**1d**	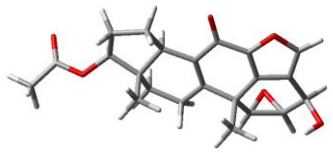	-1304.305629	-818464.1728	30.70

Table S2. Cartesian coordinates of all conformers of (1*R*, 2*S*, 3*R*, 10*R*, 13*S*, 14*R*, 17*S*)-**1**

(1*R*, 2*S*, 3*R*, 10*R*, 13*S*, 14*R*, 17*S*)-**1a**						
NO	LB	ZA	MASS	X	Y	Z
1	C	6	12.011	2.026797	-0.823083	0.279885
2	C	6	12.011	2.833992	0.413817	0.154267
3	C	6	12.011	2.320482	1.668172	0.102134
4	C	6	12.011	0.904519	1.959822	-0.018098
5	C	6	12.011	0.044073	0.729588	-0.089674
6	C	6	12.011	0.559848	-0.536081	-0.083325
7	C	6	12.011	2.738053	-1.903024	-0.550638
8	C	6	12.011	4.199252	-1.851056	-0.758884
9	C	6	12.011	5.074235	-0.721769	-0.206913
10	C	6	12.011	4.23687	0.511309	-0.047433
11	C	6	12.011	-1.44367	0.936267	-0.335953
12	C	6	12.011	-2.291741	-0.286828	0.101785
13	C	6	12.011	-1.777257	-1.507082	-0.661642
14	C	6	12.011	-0.294918	-1.767936	-0.324097
15	C	6	12.011	-2.217524	2.141593	0.255743
16	C	6	12.011	-3.709865	1.707365	0.139259
17	C	6	12.011	-3.682461	0.224931	-0.309958
18	C	6	12.011	4.483352	1.581487	-0.186332
19	O	8	15.999	3.333057	2.758079	-0.088038
20	O	8	15.999	0.460378	3.104959	-0.134975
21	O	8	15.999	-4.72261	-0.57628	0.301278
22	C	6	12.011	-5.94894	-0.536763	0.25886
23	C	6	12.011	-6.907535	-1.440771	-0.477984
24	O	8	15.999	-6.228807	0.139894	-1.230185
25	O	8	15.999	3.291866	1.529121	-1.825288
26	H	1	1.008	-1.56665	-1.017581	1.428949
27	O	8	15.999	5.572775	-1.237451	1.036996
28	C	6	12.011	2.076861	-1.314956	1.766614
29	C	6	12.011	-2.24669	-0.539438	1.621055
30	H	1	1.008	2.274058	-2.888331	-0.541182
31	H	1	1.008	4.732962	-2.793128	-0.884173
32	H	1	1.008	5.907878	-0.539403	-0.898988
33	H	1	1.008	-2.36462	-2.404257	-0.432302
34	H	1	1.008	-1.882794	-1.324292	-1.739012
35	H	1	1.008	0.157337	-2.346199	-1.140297
36	H	1	1.008	-0.223762	-2.42122	0.556304
37	H	1	1.008	-2.00997	3.062143	-0.287553
38	H	1	1.008	-1.93774	2.317216	1.299789
39	H	1	1.008	-4.229837	1.787417	1.099389
40	H	1	1.008	-4.264563	2.317996	-0.578154

41	H	1	1.008	-3.806491	0.148762	-1.393937
42	H	1	1.008	5.381654	2.424091	-0.366646
43	H	1	1.008	-6.546688	2.474063	0.449183
44	H	1	1.008	-6.969599	-1.144417	1.530022
45	H	1	1.008	-7.895023	-1.38249	0.01918
46	H	1	1.008	6.161584	-0.56422	1.415047
47	H	1	1.008	3.118989	-1.447429	2.069905
48	H	1	1.008	1.606229	-0.577697	2.422675
49	H	1	1.008	1.555762	-2.270578	1.880952
50	H	1	1.008	-1.223264	-0.690436	1.978376
51	H	1	1.008	-2.669216	0.292772	2.195357
52	H	1	1.008	-2.827215	-1.433842	1.871715

(1*R*, 2*S*, 3*R*, 10*R*, 13*S*, 14*R*, 17*S*)-**1b**						
NO	LB	ZA	MASS	X	Y	Z
1	C	6	12.011	1.90763	-0.80879	0.339799
2	C	6	12.011	2.792471	0.363806	0.141044
3	C	6	12.011	2.360696	1.643424	0.014762
4	C	6	12.011	0.966508	2.018775	-0.124318
5	C	6	12.011	0.028058	0.844741	-0.122922
6	C	6	12.011	0.460734	-0.448911	-0.03987
7	C	6	12.011	2.544843	-1.97961	-0.425641
8	C	6	12.011	4.005601	-2.033586	-0.63819
9	C	6	12.011	4.95388	-0.932544	-0.154307
10	C	6	12.011	4.197923	0.358744	-0.065901
11	C	6	12.011	-1.444248	1.133001	-0.384042
12	C	6	12.011	-2.373506	-0.003622	0.119332
13	C	6	12.011	-1.934877	-1.2965	-0.570217
14	C	6	12.011	-0.47457	-1.63281	-0.205844
15	C	6	12.011	-2.132912	2.419804	0.134311
16	C	6	12.011	-3.650054	2.085329	0.028373
17	C	6	12.011	-3.73134	0.581505	-0.325389
18	C	6	12.011	4.529855	1.669644	-0.283702
19	O	8	15.999	3.428906	2.473313	-0.228738
20	O	8	15.999	0.598445	3.181734	-0.310346
21	O	8	15.999	-4.872468	-0.005831	0.348572
22	C	6	12.011	-5.565768	-0.978177	-0.280826
23	C	6	12.011	-6.728789	-1.43629	0.566001
24	O	8	15.999	-5.287859	-1.416159	-1.381023
25	O	8	15.999	3.115331	-1.717386	-1.72061
26	H	1	1.008	-1.56294	1.158775	-1.480392
27	O	8	15.999	5.426066	-1.405704	1.116359
28	C	6	12.011	1.931676	-1.214818	1.85276

29	C	6	12.011	-2.351346	-0.1715	1.654064
30	H	1	1.008	2.019187	-2.931207	-0.357491
31	H	1	1.008	4.477344	-3.013659	-0.708723
32	H	1	1.008	5.793434	-0.844065	-0.857572
33	H	1	1.008	-2.578162	-2.141262	-0.298674
34	H	1	1.008	-2.023721	-1.169193	-1.656466
35	H	1	1.008	-0.061396	-2.29318	-0.979397
36	H	1	1.008	-0.449441	-2.230244	0.715935
37	H	1	1.008	-1.857731	3.292874	-0.455071
38	H	1	1.008	-1.8472	2.631121	1.169724
39	H	1	1.008	-4.171003	2.266161	0.973671
40	H	1	1.008	-4.151762	2.689851	-0.733417
41	H	1	1.008	-3.867293	0.426601	-1.398022
42	H	1	1.008	5.462197	2.171578	-0.49762
43	H	1	1.008	-6.365347	-1.826339	1.522327
44	H	1	1.008	-7.389319	-0.591624	0.786453
45	H	1	1.008	-7.284308	-2.212793	0.03922
46	H	1	1.008	6.063007	-0.753336	1.450445
47	H	1	1.008	2.963797	-1.400143	2.162542
48	H	1	1.008	1.515158	-0.410245	2.464847
49	H	1	1.008	1.347516	-2.124422	2.023117
50	H	1	1.008	-1.341533	-0.365771	2.024957
51	H	1	1.008	-2.725435	0.714924	2.174426
52	H	1	1.008	-2.987194	-1.013833	1.945826

(1*R*, 2*S*, 3*R*, 10*R*, 13*S*, 14*R*, 17*S*)-**1c**						
NO	LB	ZA	MASS	X	Y	Z
1	C	6	12.011	1.901749	-0.794654	0.39319
2	C	6	12.011	2.794812	0.362997	0.136635
3	C	6	12.011	2.361631	1.643472	0.011936
4	C	6	12.011	0.965401	2.021967	-0.107548
5	C	6	12.011	0.024988	0.849167	-0.103919
6	C	6	12.011	0.457397	-0.4429	-0.00816
7	C	6	12.011	2.52778	-2.011514	-0.301083
8	C	6	12.011	3.982825	-2.068686	-0.565844
9	C	6	12.011	4.95946	-0.931051	-0.235852
10	C	6	12.011	4.195055	0.35425	-0.109484
11	C	6	12.011	-1.444807	1.134011	-0.380503
12	C	6	12.011	-2.377072	-0.00186	0.119448
13	C	6	12.011	-1.92907	-1.298188	-0.557909
14	C	6	12.011	-0.471949	-1.63029	-0.176655
15	C	6	12.011	-2.140549	2.422196	0.124418
16	C	6	12.011	-3.655829	2.084815	0.001973

17	C	6	12.011	-3.731306	0.578372	-0.342591
18	C	6	12.011	4.521383	1.665441	-0.342266
19	O	8	15.999	3.424212	2.468748	-0.265043
20	O	8	15.999	0.598588	3.185955	-0.286607
21	O	8	15.999	-4.878708	-0.005139	0.323967
22	C	6	12.011	-5.559839	-0.988182	-0.30212
23	C	6	12.011	-6.735019	-1.436965	0.532837
24	O	8	15.999	-5.264998	-1.43952	-1.392448
25	O	8	15.999	3.041234	-1.841639	-1.631555
26	H	1	1.008	-1.552565	1.154098	-1.478099
27	O	8	15.999	5.734741	-1.282005	0.915847
28	C	6	12.011	1.89563	-1.114173	1.926258
29	C	6	12.011	-2.370722	-0.160973	1.655309
30	H	1	1.008	2.016696	-2.960381	-0.140832
31	H	1	1.008	4.459238	-3.049197	-0.585406
32	H	1	1.008	5.692146	-0.861917	-1.04648
33	H	1	1.008	-2.574042	-2.142137	-0.288328
34	H	1	1.008	-2.006136	-1.177406	-1.645753
35	H	1	1.008	-0.047289	-2.29191	-0.943057
36	H	1	1.008	-0.454931	-2.224177	0.747808
37	H	1	1.008	-1.859659	3.293263	-0.465268
38	H	1	1.008	-1.867204	2.637851	1.162215
39	H	1	1.008	-4.188745	2.271466	0.939413
40	H	1	1.008	-4.148571	2.683434	-0.770214
41	H	1	1.008	-3.8564	0.41606	-1.415486
42	H	1	1.008	5.448744	2.164048	-0.583413
43	H	1	1.008	-6.389995	-1.785511	1.511565
44	H	1	1.008	-7.414703	-0.596154	0.705418
45	H	1	1.008	-7.265093	-2.240449	0.020501
46	H	1	1.008	5.165139	-1.199848	1.698315
47	H	1	1.008	2.908788	-1.341198	2.272316
48	H	1	1.008	1.520954	-0.256195	2.48981
49	H	1	1.008	1.265798	-1.982036	2.143492
50	H	1	1.008	-1.364513	-0.350844	2.038062
51	H	1	1.008	-2.752162	0.727528	2.166657
52	H	1	1.008	-3.007724	-1.003105	1.945068

(1*R*, 2*S*, 3*R*, 10*R*, 13*S*, 14*R*, 17*S*)-**1d**						
NO	LB	ZA	MASS	X	Y	Z
1	C	6	12.011	2.021056	-0.81396	0.33364
2	C	6	12.011	2.837381	0.412042	0.150326
3	C	6	12.011	2.322864	1.667235	0.101489
4	C	6	12.011	0.904796	1.961449	0.002011

5	C	6	12.011	0.041733	0.732775	-0.066946
6	C	6	12.011	0.556336	-0.53252	-0.047846
7	C	6	12.011	2.721534	-1.943158	-0.433781
8	C	6	12.011	4.17677	-1.891306	-0.698846
9	C	6	12.011	5.079316	-0.716904	-0.294562
10	C	6	12.011	4.235095	0.507452	-0.092037
11	C	6	12.011	-1.443575	0.937619	-0.328246
12	C	6	12.011	-2.295231	-0.282945	0.110128
13	C	6	12.011	-1.773368	-1.509133	-0.63909
14	C	6	12.011	-0.293699	-1.767002	-0.287597
15	C	6	12.011	-2.223836	2.146804	0.246457
16	C	6	12.011	-3.714609	1.711194	0.115759
17	C	6	12.011	-3.681912	0.224752	-0.320008
18	C	6	12.011	4.476594	1.848456	-0.244401
19	O	8	15.999	3.330229	2.574029	-0.122135
20	O	8	15.999	0.462501	3.107577	-0.10719
21	O	8	15.999	-4.72804	-0.571	0.287797
22	C	6	12.011	-5.946712	-0.54326	-0.289826
23	C	6	12.011	-6.91282	-1.439421	0.44672
24	O	8	15.999	-6.215188	0.119284	-1.273965
25	O	8	15.999	3.220434	-1.658562	-1.750069
26	H	1	1.008	-1.555611	1.010567	-1.423015
27	O	8	15.999	5.874614	-1.090402	0.836219
28	C	6	12.011	2.039777	-1.227226	1.843942
29	C	6	12.011	-2.265454	-0.524192	1.635509
30	H	1	1.008	2.271888	-2.930545	-0.333597
31	H	1	1.008	4.714198	-2.836567	-0.778079
32	H	1	1.008	5.806998	-0.550702	-1.095507
33	H	1	1.008	-2.362447	-2.404577	-0.40765
34	H	1	1.008	-1.869437	-1.335038	-1.718721
35	H	1	1.008	0.167604	-2.348257	-1.096714
36	H	1	1.008	-0.228729	-2.415696	0.596882
37	H	1	1.008	-2.010365	3.063883	-0.300511
38	H	1	1.008	-1.956089	2.329508	1.291883
39	H	1	1.008	-4.246053	1.799912	1.068785
40	H	1	1.008	-4.260507	2.315297	-0.613866
41	H	1	1.008	-3.794945	0.139024	-1.404423
42	H	1	1.008	5.369877	2.418813	-0.452975
43	H	1	1.008	-6.543216	-2.469948	0.44851
44	H	1	1.008	-6.998878	-1.120282	1.490442
45	H	1	1.008	-7.89123	-1.399173	-0.032956
46	H	1	1.008	5.299213	-1.102246	1.618709
47	H	1	1.008	3.066486	-1.409414	2.176429

48	H	1	1.008	1.61251	-0.431535	2.459179
49	H	1	1.008	1.467761	-2.145662	2.006409
50	H	1	1.008	-1.245647	-0.672395	2.000349
51	H	1	1.008	-2.693766	0.312041	2.195535
52	H	1	1.008	-2.848234	-1.416909	1.882862

3. The physicochemical data of the known compounds 2-16, 18-19 and 21-22

Secalonic acid D (**2**): yellow oil; $[\alpha]_D^{25}$ = +73.8 (*c* = 0.1, $CHCl_3$); ^1H NMR (500 MHz, Acetone-*d*$_6$) and ^{13}C NMR (125 MHz, Acetone-*d*$_6$) , Table S3.

Table S3. ^1H NMR and ^{13}C NMR Data of Compound **2** and Comparison with Literature Values

	Secalonic acid D (**2**) (Acetone-*d*$_6$)		Reference ($CDCl_3$) [1]	
Position	δ_H, mult. (*J* in Hz)	δ_C	δ_H, mult. (*J* in Hz)	δ_C
2		118.59		118.2
3	7.49, d (8.5)	141.23	7.46, dd (8.2)	140.2
4	6.58, d (8.5)	107.49	6.62, d (8.2)	106.9
4a		160.20		158.3
5	3.97, dd (10.8)	77.10	3.93, d (10.6)	77.2
6	2.47, m	30.73	2.42, m	29.2
7	(a) 2.46, dd	36.80	(a) 2.32, dd (18.8, 10.6)	36.2
	(b) 2.73, dd		(b) 2.74, dd (18.8, 5.8)	
8a		102.87		101.5
9		179.48		177.5
9a		108.38		107.6
10a		86.18		84.7
11	1.16, d (6.2)	18.28	1.17, d (6.3)	18.0
12		171.04		170.3
13	3.65, s	53.04	3.73, s	53.3
1-OH	11.64, s	160.31	11.8, s	159.4
8-OH	13.77, s	188.52	13.8, s	187.2
2′	---	118.59	---	118.2
3′	7.49, d (8.5)	141.23	7.46, dd (8.2)	140.2
4′	6.58, d (8.5)	107.49	6.62, d (8.2)	106.9
4a′		160.20		158.3
5′	3.97, dd (10.8)	77.10	3.93, d (10.6)	77.2
6′	2.47, m	30.73	2.42, m	29.2
7′	(a) 2.46, dd	36.80	(a) 2.32, dd (18.8, 10.6)	36.2
	(b) 2.73, dd		(b) 2.74, dd (18.8, 5.8)	
8a′		102.87		101.5
9′		179.48		177.5
9a′		108.38		107.6
10a′		86.18		84.7
11′	1.16, d (6.2)	18.28	1.17, d (6.3)	18.0

12′		171.04		170.3
13′	3.65, s	53.04	3.73, s	53.3
1′-OH	11.64, s	160.31	11.8, s	159.4
8′-OH	13.77, s	188.52	13.8, s	187.2

Penicillixanthone A (**3**): yellow solid; ^{1}H NMR (500 MHz, CD_3OD) and ^{13}C NMR (125 MHz, CD_3OD), Table S4.， Its ^{1}H and ^{13}C NMR data were consistent with those reported for penicillixanthone A [2].

Table S4. ^{1}H NMR and ^{13}C NMR Data of Compound **3** and Comparison with Literature Values

	Penicillixanthone A (**3**) (CD_3OD)		Reference (Acetone-d_6) [2]	
Position	δ_H, mult. (*J* in Hz)	δ_C	δ_H, mult. (*J* in Hz)	δ_C
2		119.50		118.01
3	7.56, d (8.5)	142.31	7.81, d (8.7)	140.69
4	6.55, d (8.5)	108.19	6.63, d (8.7)	107.37
4a		158.99		158.26
5	3.82, dd (5.1)	77.38	3.944, d (10.8)	76.61
6	2.46, m	30.73	2.43, m	29.28
7	(a) 2.70, m	36.80	(a) 2.28, m	36.24
	(b) 2.43, m		(b) 2.75, m	
8a		103.43		101.65
9		189.49		187.18
9a		107.93		106.95
10a		86.58		84.90
11	1.08, d (6.2)	18.28	1.19, d (6.6)	18.01
12		173.32		170.30
13	3.63, s	53.91	3.73, s	53.29
1-OH	11.34, s	160.33	11.65, s	159.33
8-OH	13.76, s	180.63	13.76, s	177.66
2′	---	110.38	---	110.41
3′	7.99, d (8.5)	142.10	7.50, d (8.7)	140.65
4′	6.55, d (8.5)	116.87	6.61, d (8.7)	115.42
4a′		157.75		155.25
5′	3.93, dd (10.8)	72.36	3.84, d (10.8)	76.81
6′	2.46, m	30.73	2.42, m	29.06
7′	(a) 2.43, m	36.80	(a) 2.33, m	36.20
	(b) 2.70, m		(b) 2.71, m	
8a′		101.67		101.47
9′		188.96		187.11
9a′		108.63		107.47
10a′		86.39		84.83
11′	1.15, d (6.2)	17.85	1.12, d (6.0)	17.92
12′		172.02		170.12

13′	3.63, s	53.34	3.68, s	53.21
1′-OH	11.62, s	162.70	11.39, s	161.71
8′-OH	13.76, s	178.74	13.71, s	177.29

2,2′,6′-Trihydroxy-4-methyl-6-methoxy-acyl-diphenylmethanone (**4**): yellow powder; ^{1}H NMR (700 MHz, CD_3OD) and ^{13}C NMR (176 MHz, CD_3OD) , Table S5. Its ^{1}H and ^{13}C NMR data were consistent with those reported for 2,2′,6′-trihydroxy-4-methyl-6-methoxy-acyl-diphenylmethanone [3].

Table S5. ^{1}H NMR and ^{13}C NMR Data of Compound **4** and Comparison with Literature Values

	2,2′,6′-Trihydroxy-4-methyl-6-methoxy-acyl-diphenylmethanone (**4**) (CD_3OD)		Reference (DMSO-d_6) [3]	
Position	δ_H, mult. (J in Hz)	δ_C	δ_H, mult. (J in Hz)	δ_C
1		131.83		130.3
2		154.84		153.4
3	6.87, brs	121.47	6.89, brs	120.4
4		140.34		138.4
5	7.29, brs	122.19	7.20, brs	120.3
6		129.29		127.4
7		168.35		166.0
8	2.33, s	21.22	2.29, s	20.19
9	3.67, s	52.39	3.65, s	51.9
1′		112.79		111.3
2′, 6′		163.39		161.0
3′, 5′	6.28, d (8.2)	108.04	6.25, d (8.1)	106.8
4′	7.20, t (8.2)	137.33	7.22, d (8.1)	136.3
7′		203.28		201.0
2-OH			9.8, brs	
2′-OH			11.4, brs	
6′-OH			11.4, brs	

Sclerin diacid (**5**): yellow oil; ^{1}H NMR (700 MHz, CD_3OD) and ^{13}C NMR (176 MHz, CD_3OD), Table S6. Its ^{1}H and ^{13}C NMR data were consistent with those reported for sclerin diacid [4].

Table S6. ^{1}H NMR and ^{13}C NMR Data of Compound **5** and Comparison with Literature Values

	Sclerin diacid (**5**) (CD_3OD)		Reference (CD_3OD) [4]	
Position	δ_H, mult. (J in Hz)	δ_C	δ_H, mult. (J in Hz)	δ_C
1		174.49		172.94
3		177.45		176.36
4	4.44, q (7.0)	43.24	4.416, q (7.4)	42.01
5		138.73		137.43
6		127.54		126.39

7		143.49		142.84
8		124.41		123.56
9		158.45	10.754, s	157.54
10		112.37		109.98
11	1.52, d (6.9)	17.44	1.570, d (7.4)	17.12
12	2.24, s	17.36	2.306, s	16.61
13	2.13, s	16.39	2.125, s	15.94
14	2.19, s	12.36	2.189, s	11.91

Sclerin diacid monoester (**6**): yellow oil; ^{1}H NMR (700 MHz, CD_3OD) and ^{13}C NMR (176 MHz, CD_3OD), Table S7. Its ^{1}H and ^{13}C NMR data were consistent with those reported for sclerin diacid monoester [4].

Table S7. ^{1}H NMR and ^{13}C NMR Data of Compound **6** and Comparison with Literature Values

	Sclerin diacid monoester (**6**) (CD_3OD)		Reference ($CDCl_3$) [4]	
Position	δ_H, mult. (*J* in Hz)	δ_C	δ_H, mult. (*J* in Hz)	δ_C
1		178.37		179.91
3		172.86		171.40
4	4.07, q (8.8)	43.47	4.315, q (7.6)	42.22
5		138.23		136.17
6		124.40		123.88
7		143.28		143.40
8		127.97		126.75
9		156.81	10.767, s	157.76
10		113.37		109.52
11	1.42, d (9.1)	21.41	1.526, d (7.6)	22.10
12	2.11, s	16.42	2.240, s	16.70
13	2.13, s	17.43	2.283, s	16.83
14	2.18, s	12.41	2.240, s	12.38
OMe	3.70, s	51.88	3.871, s	51.36

(3R,4S)-6,8-Dihydroxy-3,4,7-trimethylisocoumarin (**7**): white powder; ^{1}H NMR (500 MHz, DMSO-d_6) and ^{13}C NMR (125 MHz, DMSO-d_6), Table S8. Its ^{1}H and ^{13}C NMR data were consistent with those reported for (3R,4S)-6,8-dihydroxy-3,4,7-trimethylisocoumarin [5].

Table S8. ^{1}H NMR and ^{13}C NMR Data of Compound **7** and Comparison with Literature Values

	(3R,4S)-6,8-Dihydroxy-3,4,7-trimethylisocoumarin (**7**) (DMSO-d_6)		Reference (DMSO-d_6) [5]	
Position	δ_H, mult. (*J* in Hz)	δ_C	δ_H, mult. (*J* in Hz)	δ_C
1		113.2		113.2
2		160.3		160.2
3		108.4		108.4
4		159.1		159.1

4a		98.7		98.6
5		169.7		169.7
7		104.6		104.6
8	3.11, d (7.0)	38.3	3.11, q (7.0)	38.2
8a		141.3		141.2
1-CH_3	2.03, s	10.6	2.04, s	10.6
3-CH_3	2.02, s	8.37	2.02, s	8.3
7-CH_3	1.59, s	25.1	1.59, s	25.1
8-CH_3	1.05, d (7.0)	17.1	1.04, d (7.1)	17.1
2-OH	9.37, brs		9.33, s	
4-OH	11.67, brs		11.67, s	
7-OH	7.27, brs		7.25, s	

2,4-Dihydroxy-3-methylacetophenone (**8**): yellow powder; ^{1}H NMR (500 MHz, Acetone-d_6) and ^{13}C NMR (125 MHz, Acetone-d_6), Table S9. Its ^{1}H and ^{13}C NMR data were consistent with those reported for 2,4-dihydroxy-3-methylacetophenone [6].

Table S9. ^{1}H NMR and ^{13}C NMR Data of Compound **8** and Comparison with Literature Values

	2,4-Dihydroxy-3-methylacetophenone (**8**) (Acetone-d_6)		Reference (CD_3OD) [6]	
Position	δ_H, mult. (J in Hz)	δ_C	δ_H, mult. (J in Hz)	δ_C
1		113.8		113.1
2		163.5		164.1
3		111.6		113.9
4		163.0		164.3
5	6.51, d (8.7)	107.7	6.43, d (8.8)	108.1
6	7.62, d (8.7)	130.9	7.62, d (8.9)	131.1
7		203.8		204.3
8	2.53, s	26.3	2.57, s	26.1
9	2.04, s	7.6	2.08, s	7.5

2,4-Dihydroxy-5-methylacetophenone (**9**): yellow powder; ^{1}H NMR (500 MHz, Acetone-d_6) and ^{13}C NMR (125 MHz, Acetone-d_6), Table S10. Its ^{1}H and ^{13}C NMR data were consistent with those reported for 2,4-dihydroxy-5-methylacetophenone [7].

Table S10. ^{1}H NMR and ^{13}C NMR Data of Compound **9** and Comparison with Literature Values

	2,4-Dihydroxy-5-methylacetophenone (**9**) (Acetone-d_6)		Reference (CD_3OD) [7]	
Position	δ_H, mult. (J in Hz)	δ_C	δ_H, mult. (J in Hz)	δ_C
1		133.1		134.1
2	7.64, s	112.9	7.46, s	114.0
3		116.4		118.2

4		162.9		164.7
5	6.37, s	101.9	6.33, s	102.8
6		163.1		164.5
7		202.6		204.1
8	2.53, s	25.4	2.55, s	26.2
9	2.13, s	14.5	2.19, s	15.5

4-Acetyl-resorcinol (**10**): brown oil; ^{1}H NMR (500 MHz, Acetone-*d*$_6$) and ^{13}C NMR (125 MHz, Acetone-*d*$_6$) , Table S11. Its ^{1}H and ^{13}C NMR data were consistent with those reported for 4-acetyl-resorcinol [8].

Table S11. ^{1}H NMR and ^{13}C NMR Data of Compound **10** and Comparison with Literature Values

	4-Acetyl-resorcinol (**10**) (Acetone-*d*$_6$)		Reference (CD_3OD) [8]	
Position	δ_H, mult. (*J* in Hz)	δ_C	δ_H, mult. (*J* in Hz)	δ_C
1		114.1		112.8
2		165.8		164.8
3	6.33, s (2.3)	103.3	6.24, d (2.5)	102.2
4		165.6		164.2
5	6.44, dd (8.8, 2.3)	108.7	6.38, dd (8.9, 2.3)	108.1
6	7.77, d (8.8)	134.3	7.76, d (8.9)	133.7
7		203.6		202.7
8	2.61, s	26.3	2.52, s	26.3

6-(1-Hydroxy-2-methylpropyl)-3-(2-methylpropyl)-2(1H) pyrazinone (**11**): yellow powder; $[\alpha]^{25}_{D}$ = -1.5 (*c* = 0.1, CH_3OH); ^{1}H NMR (500 MHz, DMSO-*d*$_6$) and ^{13}C NMR (125 MHz, DMSO-*d*$_6$) , Table S12. Its ^{1}H and ^{13}C NMR data were consistent with those reported for 6-(1-hydroxy-2-methylpropyl)-3-(2-methylpropyl)-2(1H) pyrazinone [9].

Table S12. ^{1}H NMR and ^{13}C NMR Data of Compound **11** and Comparison with Literature Values

	6-(1-Hydroxy-2-methylpropyl)-3-(2-methylpropyl)-2(1H) pyrazinone (**11**) (DMSO-*d*$_6$)		Reference ($CDCl_3$) [9]	
Position	δ_H, mult. (*J* in Hz)	δ_C	δ_H, mult. (*J* in Hz)	δ_C
2		156.12		157.3
3		156.12		158.46
5	7.20, s	124.64	7.31, s	121.78
6				138.44
7	4.05, d (6.4)	73.03	4.31, d (6)	74.30
8	1.95, m	32.74	1.96, dqq (6, 6.5, 6.5)	34.19
9	0.78, d (6.8)	18.94	0.90, d (6.5)	18.92
10	0.87, d	17.41	0.99, d (6.5)	17.70
11	2.48, dd	40.11	(a) 2.64, dd (7, 14)	41.64
	2.13, dd		(b) 2.73, dd (7, 14)	

12	2.10, dt (6.8)	26.07	2.21, tqq (7, 7, 7)	26.88
13	0.85, d (6.7)	22.47	0.95, d (7)	22.59
14	0.85, d (6.7)	22.47	0.95, d (7)	22.64

Insulicolide A (**12**): white powder; ^{1}H NMR (500 MHz, CD_3OD) and ^{13}C NMR (125 MHz, CD_3OD) , Table S13. Its ^{1}H and ^{13}C NMR data were consistent with those reported for insulicolide A [10].

Table S13. ^{1}H NMR and ^{13}C NMR Data of Compound **12** and Comparison with Literature Values

	Insulicolide A (**12**) (CD_3OD)		Reference (CD_3OD) [10]	
Position	δ_H, mult. (J in Hz)	δ_C	δ_H, mult. (J in Hz)	δ_C
1	1.38, m	33.3	1.37, m	33.3
	2.17, m		2.17, m	
2	1.57, m	18.7	1.57, m	18.7
	1.72, m		1.72, m	
3	1.08, m	36.9	1.01, m	36.9
	2.24, m		2.24, m	
4		40.5		40.5
5	2.49, d (4.6)	48.3	2.49, d (4.6)	48.0
6	6.14, dd (4.6, 4.0)	65.5	6.14, dd (4.6, 4.0)	65.5
7	6.80, d (4.0)	133.3	6.79, d (4.0)	133.3
8		134.9		134.9
9		77.8		77.8
10		40.5		40.5
11	4.57, d (9.9)	76.4	4.57, d (9.9)	76.4
	4.26, d (9.9)		4.27, d (9.9)	
12		171.1		171.0
13	1.13, s	27.4	1.12, s	27.4
14	4.14, d (11.1)	69.1	4.14, d (11.1)	69.0
	3.53, d (11.1)		3.53, d (11.1)	
15	1.36, s	21.9	1.36, s	21.9
1′		165.2		165.2
2′		136.4		136.4
3′, 7′	8.25, brd (8.9)	132.1	8.25, brd (8.9)	132.1
4′, 6′	8.39, brd (8.9)	125.2	8.39, brd (8.9)	125.0
5′		152.4		152.4

6β,9α-Dihydroxy-14-p-nitrobenzoylcinnamolide (**13**): white powder; ^{1}H NMR (500 MHz, CD_3OD) and ^{13}C NMR (125 MHz, CD_3OD) , Table S14. Its ^{1}H and ^{13}C NMR data were consistent with those reported for 6β,9α-dihydroxy-14-p-nitrobenzoylcinnamolide [10].

Table S14. ^{1}H NMR and ^{13}C NMR Data of Compound **13** and Comparison with Literature Values

	6β,9α-Dihydroxy-14-p-		Reference (CD_3OD) [10]	

Position	nitrobenzoylcinnamolide (**13**) (CD_3OD)			
	δ_H, mult. (*J* in Hz)	δ_C	δ_H, mult. (*J* in Hz)	δ_C
1	1.30, m	31.5	1.29, m	31.5
	2.08, m		2.08, m	
2	1.57, m	17.4	1.57, m	17.3
	1.75, m		1.75, m	
3	1.19, m	36.3	1.17, m	36.3
	2.16, m		2.16, m	
4		38.6		38.6
5	2.21, d (4.6)	47.9	2.20, d (4.6)	47.9
6	4.72, dd (4.6, 4.0)	63.6	4.72, dd (4.6, 4.0)	63.6
7	6.82, d (4.0)	139.0	6.82, d (4.0)	138.9
8		129.4		129.4
9		76.6		76.6
10		38.1		38.1
11	4.55, d (9.9)	75.1	4.55, d (9.9)	75.1
	4.23, d (9.9)		4.23, d (9.9)	
12		170.5		170.5
13	1.22, s	26.1	1.20, s	26.1
14	5.18, d (11.4)	68.6	5.18, d (11.4)	68.6
	5.02, d (11.4)		5.02, d (11.4)	
15	1.35, s	20.0	1.35, s	19.9
1′		164.9		164.9
2′		135.9		135.9
3′, 7′	8.26, brd (8.9)	130.4	8.26, brd (8.9)	130.3
4′, 6′	8.39, brd (8.9)	123.3	8.38, brd (8.9)	123.3
5′		150.7		150.7

Pre-sclerotiotide F (**14**): white powder; ^1H NMR (500 MHz, CD_3OD) and ^{13}C NMR (125 MHz, CD_3OD) , Table S15. Its ^1H and ^{13}C NMR data were consistent with those reported for pre-sclerotiotide [11].

Table S15. ^1H NMR and ^{13}C NMR Data of Compound **14** and Comparison with Literature Values

Position	Pre-sclerotiotide F (**14**) (CD_3OD)		Reference ($CDCl_3$) [11]	
	δ_H, mult. (*J* in Hz)	δ_C	δ_H, mult. (*J* in Hz)	δ_C
1		175.06		173.1
2	5.03, ddd (10.7)	51.43	5.05, ddd (7.2, 7.2, 0.6)	49.7
3	2.28, m, 1.79, m	29.13	2.37, m, 1.71, m	28.2
4	1.86, m, 1.57, m	23.10	1.76, m, 1.58, m	21.8
5	3.31, m, 3.04, m	49.35	3.48, m, 2.95, m	47.9
6	4.29, dq (6.8)	40.96	4.72, dd (4.6, 4.0)	39.9
7	6.63, brd (7.7)		6.44, brd (6.0)	
8		173.95		172.5

9	4.93, d (7.7)	53.03	4.19, dq (9.0, 7.2)	51.7
10	1.38, d (7.2)	19.17	1.45, d (7.2)	19.3
11		172.83		170.8
12	5.03, d (10.7)	58.98	4.98, d (10.2)	58.4
13	2.32, m	27.51	2.39, m	26.3
14	0.77, d (6.6)	18.21	0.75, d (6.6)	17.8
15	0.95, d (6.0)	19.87	0.96, d (6.6)	19.7
17	7.35, m		7.20, d (9.0)	
18	3.10, s	30.59	3.00, s	30.3
19	7.15, d (7.2)		7.04, d (7.2)	
20		166.11		163.4
21	6.62, d (14.8)	133.55	6.40, d (15.0)	132.0
22	7.37, m	138.50	7.38, dd (15.0, 11.4)	137.5
23	7.32, m	150.06	7.13, dd (15.0,11.4)	147.5
24	6.41, dd (14.8, 7.7)	137.46	6.41, dd (15.0, 8.4)	136.5
25	9.63, d (7.8)	195.43	9.65, d (8.4)	193.0

Wortmannolone (**15**): colorless crystals; ^{1}H NMR (500 MHz, CD_3OD) and ^{13}C NMR (125 MHz, CD_3OD) , Table S16. Its ^{1}H and ^{13}C NMR data were consistent with those reported for wortmannolone [12].

Table S16. ^{1}H NMR and ^{13}C NMR Data of Compound **15** and Comparison with Literature Values

	Wortmannolone (**15**) (CD_3OD)		Reference (CD_3OD) [12]	
Position	δ_H, mult. (J in Hz)	δ_C	δ_H, mult. (J in Hz)	δ_C
1	3.72, d (4.0)	55.6	3.70, d (4.0)	55.7
2	3.47, dd (4.0, 2.4)	54.8	3.47, dd (4.0, 2.4)	54.7
3	5.23, d (2.4)	60.9	5.23, d (2.4)	60.9
4		121.8		121.8
5		143.1		143.0
6		146.2		146.2
7		175.8		175.8
8		135.1		135.1
9		162.0		162.0
10		42.4		42.4
11	2.85, m	25.3	2.85, m, 3.05, m	25.3
12	2.00, m, 2.57, m	28.7	1.95, m, 2.57, m	28.7
13		48.9		48.9
14	2.71, dd (10.0, 2.8)	45.0	2.71, dd (10.0, 2.8)	45.0
15	2.85, m, 1.65, m	23.9	2.85, m, 1.61, m	23.9
16	2.25, m, 2.01, m	37.5	2.25, m, 2.01, m	37.5
17	-	221.5	-	221.5
18	0.91, s	14.1	0.91, s	14.1

19	1.67, s	28.7	1.67, s	28.7
20		-		-
21	-	-	-	-
22	7.81, s	147.1	7.81, s	147.1

Wortmannolol (**16**): white powder; ^{1}H NMR (500 MHz, CD_3OD) and ^{13}C NMR (125 MHz, CD_3OD), Table S17. Its ^{1}H and ^{13}C NMR data were consistent with those reported for wortmannolol [13].

Table S17. ^{1}H NMR and ^{13}C NMR Data of Compound **16** and Comparison with Literature Values

	Wortmannolol (**16**) (CD_3OD)		Reference (CD_3OD) [13]	
Position	δ_H, mult. (*J* in Hz)	δ_C	δ_H, mult. (*J* in Hz)	δ_C
1	3.72, d (3.8)	55.9	3.70, d (3.8)	55.94
2	3.35, dd (3.7, 2.7)	54.9	3.45, dd (3.7, 2.7)	54.88
3	5.22, d (2.7)	61.1	5.21, d (2.7)	61.10
4		121.9		121.95
5		143.2		143.22
6		146.8		146.76
7		176.4		176.43
8		136.6		136.63
9		161.7		161.74
10		42.3		42.28
11	2.85, m, 3.05, m	25.8	2.85, m, 3.05, m	25.78
12	1.95, m, 2.57, m	33.3	1.95, m, 2.57, m	33.31
13		44.5		44.48
14	2.71, dd (10.0, 2.8)	44.9	2.71, dd (10.0, 2.8)	44.86
15	2.85, m, 1.61, m	25.5	2.85, m, 1.61, m	25.50
16	2.25, m, 2.01, m	31.6	2.25, m, 2.01, m	31.56
17	-	80.0	-	80.03
18	0.93, s	11.2	0.91, s	11.27
19	1.67, s	28.9	1.67, s	28.97
22	7.84, s	147.1	7.81, s	147.10

5α,8α-Epidioxy-(22E,24R)-23-ergosta-6,22-dien-3β-ol (**18**): colorless crystals; $[\alpha]_D^{25}$ = -8.7 (*c* = 0.1, CH_3OH); and ^{13}C NMR (125 MHz, $CDCl_3$) , Table S18. Its ^{1}H and ^{13}C NMR data were consistent with those reported for 5α,8α-epidioxy-(22E,24R)-23-ergosta-6,22-dien-3β-ol [14].

Table S18. ^{1}H NMR and ^{13}C NMR Data of Compound **18** and Comparison with Literature Values

	5α,8α-Epidioxy-(22E,24R)-23-ergosta-6,22-dien-3β-ol (**18**) ($CDCl_3$)		Reference ($CDCl_3$) [14]	
Position	δ_H, mult. (*J* in Hz)	δ_C	δ_H, mult. (*J* in Hz)	δ_C
1	1.34–2.08, m	34.8		34.7
2	1.34–2.08, m	30.2		30.1

3	3.97, m	66.6	3.92-4.02, m	66.4
4	1.34–2.08, m	37.0		36.9
5		82.3		82.1
6	6.24, d (8.5)	135.5	6.24, d (8.4)	135.4
7	6.50, d (8.5)	130.9	6.51, d (8.5)	130.7
8		79.6		79.4
9	1.34–2.08, m	51.2		51.1
10		37.1		36.9
11	1.34–2.08, m	23.5		23.4
12	1.34–2.08, m	39.5		39.3
13		44.7		44.5
14	1.34–2.08, m	51.8		51.7
15	1.34–2.08, m	20.8		20.6
16	1.34–2.08, m	28.8		28.6
17	1.34–2.08, m	56.3		56.2
18	0.80, s	13.0	0.82, s	12.9
19	0.88, s	18.3	0.88, s	18.2
20	1.34–2.08, m	39.9		39.7
21	1.00, d (6.5)	21.0	1.00, d (6.8)	20.9
22	5.14, dd (14.0, 9.0)	135.3	5.15, dd (15.3, 8.1)	135.2
23	5.23, dd (14.0, 7.5)	132.4	5.21, dd (15.3, 7.4)	132.3
24	1.34–2.08, m	42.9		42.8
25	1.34–2.08, m	33.2		33.0
26	0.82, overlapped	19.8	0.82, s (6.8)	19.6
27	0.83, overlapped	20.1	0.83, s (6.8)	19.9
28	0.91, d (6.5)	17.7	0.91, d (6.8)	17.5

L-Tryptophan (**19**): yellow solid; $[\alpha]_D^{25}$ = +10.65 (c = 0.1, CH_3OH); and ^{13}C NMR (126 MHz, DMSO-d_6) , Table S19[15].

Table S19. ^1H NMR and ^{13}C NMR Data of Compound **19** and Comparison with Literature Values

	L-Tryptophan (**19**) (DMSO-d_6)		Reference (DMSO-d_6) [15]	
Position	δ_H, mult. (J in Hz)	δ_C	δ_H, mult. (J in Hz)	δ_C
NH	10.99, s		10.98, s	
2	7.23, s	124.7	7.22, s	124.1
3		109.9		109.57
4	7.57, d (7.8)	118.8	7.56, d (7.8)	118.26
5	6.97, t (7.4)	118.9	6.96, t (7.5)	118.36
6	7.06, t (7.5)	121.4	7.05, t (7.8)	120.86
7	7.35, d (8.0)	111.9	7.34, d (8.1)	111.32
8		136.9		136.38
9		127.5		127.3
10	3.31, dd (4.0, 15.3)	27.6	3.34, dd (3.6, 15.0)	27.16

	3.01, dd (8.5, 15.2)		2.99, dd (8.7, 15.0)	
11	3.51, d (6.9)	55.2	3.49, dd (4.2, 8.1)	54.76
COOH		171.1		170.51

5,9-Dihydroxy-2,4,6,8,10-pentamethyldodeca-2,6,10-trienal (**21**): white powder; ^{1}H NMR (500 MHz, DMSO-d_6) and ^{13}C NMR (125 MHz, DMSO-d_6) , Table S20. Its ^{1}H and ^{13}C NMR data were consistent with those reported for 5,9-dihydroxy-2,4,6,8,10-pentamethyldodeca-2,6,10-trienal [16].

Table S20. ^{1}H NMR and ^{13}C NMR Data of Compound **21** and Comparison with Literature Values

	5,9-Dihydroxy-2,4,6,8,10-pentamethyldodeca-2,6,10-trienal (**21**) (DMSO-d_6)		Reference (DMSO-d_6) [16]	
Position	δ_H, mult. (J in Hz)	δ_C	δ_H, mult. (J in Hz)	δ_C
1	9.37, s	196.2	9.37, s	196.0
2		138.4		138.4
3	6.57, d (7.7)	160.0	6.57, d (7.7)	160.0
4	2.77, q (7.7)	37.9	2.77, q (7.7)	37.9
5	3.74, d (7.7)	81.1	3.74, d (7.7)	81.0
6		136.1		136.1
7	5.25, d (9.0)	131.4	5.25, d (9.0)	131.4
8	2.46, m	36.5	2.46, m	36.5
9	3.58, d (7.1)	81.5	3.58, d (7.1)	81.5
10		138.1		138.1
11	5.35, q (6.6)	120.2	5.33, q (6.6)	120.0
12	1.53, brs	11.8	1.53, brs	11.8
13	1.65, s	9.7	1.65, s	9.7
14	0.85, d (7.7)	17.4	0.85, d (7.7)	17.4
15	1.55, s	12.2	1.55, s	12.2
16	0.73, d (6.8)	18.6	0.73, d (6.8)	18.6
17	1.51, s	13.4	1.51, s	13.4

Penicillocitrin A (**22**): yellow powder; ^{1}H NMR (500 MHz, Acetone-d_6) and ^{13}C NMR (125 MHz, Acetone-d_6) , Table S21. Its ^{1}H and ^{13}C NMR data were consistent with those reported for penicillocitrin A [17].

Table S21. ^{1}H NMR and ^{13}C NMR Data of Compound **22** and Comparison with Literature Values

	Penicillocitrin A (**22**) (DMSO-d_6)		Reference (CD_3COD+$CDCl_3$, 1:1, v/v) [17]	
Position	δ_H, mult. (J in Hz)	δ_C	δ_H, mult. (J in Hz)	δ_C
2		164.6		165.3
3	6.21, s	113.1	6.24, s	112.7

4		183.0		183.7
4a		113.1		112.9
5		140.5		140.5
6	6.25, d (2.1)	110.0	6.26, d (2.0)	109.9
7		160.8		160.7
8	6.36, d (1.7)	101.3	6.25, d (2.0)	101.1
8a		157.7		157.9
9		105.1		105.3
10	6.40, d (2.2)	94.8	6.33, d (2.4)	94.9
11		159.8		159.8
12		163.1		162.7
13		165.8		165.8
14	6.39, d (2.1)	99.76	6.23, d (2.4)	99.9
15	2.22, s	20.24	2.20, s	20.4

4. Biological evaluation

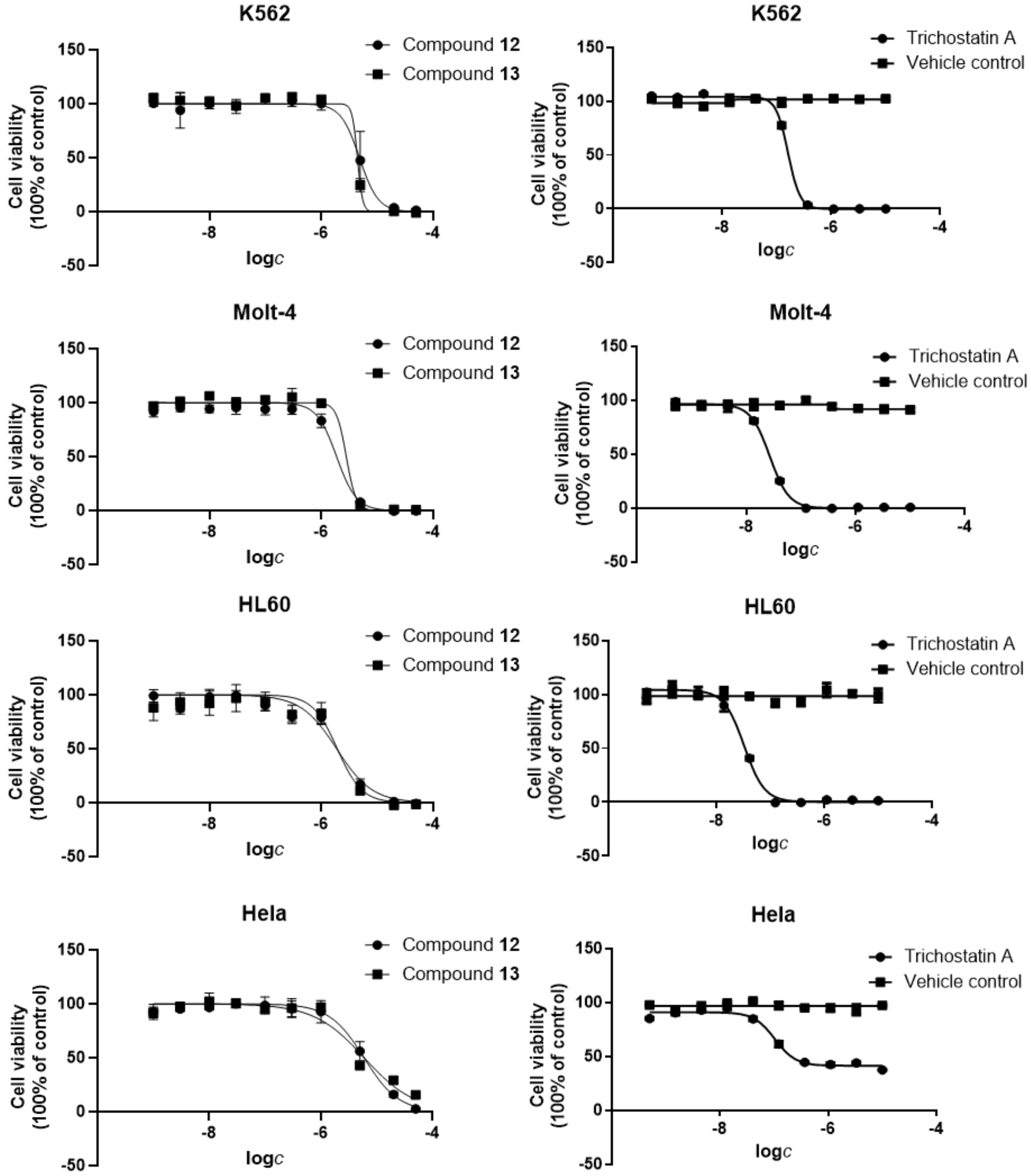

Figure S22. Cytotoxicity of compounds **12** and **13** against K562, Molt-4, HL60 and Hela cell lines

References

1. Yamazaki, H.; Ukai, K.; Namikoshi, M., Asperdichrome, an unusual dimer of tetrahydroxanthone through an ether bond, with protein tyrosine phosphatase 1B inhibitory activity, from the okinawan freshwater *Aspergillus* sp. TPU1343. *Tetrahedron Lett.* **2016,** 57, 732-735.
2. Tan, S.; Yang, B.; Liu, J.; Xun, T.; Liu, Y.; Zhou, X., Penicillixanthone A, a marine-derived dual-coreceptor antagonist as anti-HIV-1 agent. *Nat. Prod. Res.* **2019,** 33, 1467-1471.
3. Liu, B.; Wang, H.-F.; Zhang, L.-H.; Liu, F.; He, F.-J.; Bai, J.; Hua, H.-M.; Chen, G.; Pei, Y.-H., Isolation of a new compound from *Penicillium oxalicum*. *Chem. Nat. Compd.* **2016,** 52, 821-823.

4. Yuyama, K. T.; Chepkirui, C.; Wendt, L.; Fortkamp, D.; Stadler, M.; Abraham, W.-R., Bioactive compounds produced by *Hypoxylon fragiforme* against *staphylococcus aureus* biofilms. *Microorganisms* **2017,** 5, 80.
5. Lai, D.; Broetz-Oesterhelt, H.; Mueller, W. E. G.; Wray, V.; Proksch, P., Bioactive polyketides and alkaloids from *Penicillium citrinum*, a fungal endophyte isolated from *ocimum tenuiflorum*. *Fitoterapia* **2013,** 91, 100-106.
6. Gammon, D. W.; Hunter, R.; Wilson, S. A., An efficient synthesis of 7-hydroxy-2,6-dimethylchromeno[3,4-*d*]oxazol-4-one—a protected fragment of novenamine. *Tetrahedron* **2005,** 61, 10683-10688.
7. Li, J.-T.; Fu, X.-L.; Tan, C.; Zeng, Y.; Wang, Q.; Zhao, P.-J., Two New Chroman Derivations from the Endophytic *Penicillium* sp. DCS523. *Molecules* **2011**, 16, 686-693.
8. Yasuda, T.; Kon, R.; Nakazawa, T.; Ohsawa, K., Metabolism of Paeonol in Rats. *J. Nat. Prod.* **1999**, 62, 1142-1144.
9. Liu, W.; Huang, M.-y.; Tian, D.-f.; Yang, Y.; Bai, S.-k.; Wu, Y.-x.; Yu, L.-y.; Hong, B.; Jiang, W.; Si, S.-y., Isolation and structure identification of desoxyneohydroxyaspergillic acid. *Chin. J. Antibiot.* **2011,** 36, 11-13.
10. Fang, W.; Lin, X. P.; Zhou, X. F.; Wan, J. T.; Lu, X.; Yang, B.; Ai, W.; Lin, J.; Zhang, T. Y.; Tu, Z. C.; Liu, Y. H., Cytotoxic and antiviral nitrobenzoyl sesquiterpenoids from the marine-derived fungus *Aspergillus ochraceus* Jcma1F17. *Medchemcomm* **2014,** 5, 701-705.
11. Wu, Q. X.; Jin, X. J.; Draskovic, M.; Crews, M. S.; Tenney, K.; Valeriote, F. A.; Yao, X. J.; Crews, P., Unraveling the numerous biosynthetic products of the marine sediment-derived fungus, *Aspergillus insulicola*. *Phytochem. Lett.* **2012,** 5, 114-117.
12. Ding, H. E.; Yang, Z. D.; Sheng, L.; Zhou, S. Y.; Li, S.; Yao, X. J.; Zhi, K. K.; Wang, Y. G.; Zhang, F., Secovironolide, a novel furanosteroid scaffold with a five-membered B ring from the endophytic fungus *Talaromyces wortmannii* LGT-4. *Tetrahedron Lett.* **2015,** 56, 6754-6757.
13. Zhi, K. K.; Yang, Z. D.; Zhou, S. Y.; Yao, X. J.; Li, S.; Zhang, F., A new furanosteroid from *Talaromyces* sp. LGT-4, a fungal endophyte isolated from *tripterygium wilfordii*. *Nat. Prod. Res.* **2016,** 30, 2137-2141.
14. Hybelbauerová S, Sejbal J, Dracínský M, Hahnová A, Koutek B., Chemical constituents of Stereum subtomentosum and two other birch-associated basidiomycetes: an interspecies comparative study. *Chem Biodivers*. **2008,** 5, 743-50.
15. Li G. Q., Deng Z. W., Li J., Fu H. Z., Lin W. h., Chemical Constituents from Starfish *Asterias rollestoni. J. Chin. Pharm. Sci.* **20**04, 2, 81-86.
16. Tawfike, A. F.; Romli, M.; Clements, C.; Abbott, G.; Young, L.; Schumacher, M.; Diederich, M.; Farag, M.; Edrada-Ebel, R., Isolation of anticancer and anti-trypanosome secondary metabolites from the endophytic fungus *Aspergillus flocculus* via bioactivity guided isolation and MS based metabolomics. *J. Chromatogr. B Analyt. Technol. Biomed. Life Sci.* **2019,** 1106, 71-83.
17. Li, X. L.; Zhang, L.; Liu, Y. H.; Guo, Z. Y.; Deng, Z. S.; Chen, J. F.; XuanTu; Zou, K., A new metabolite from the endophytic fungus *Penicillium citrinum*. *Nat. Prod. Commun.* **2013,** 8, 587-588.
